# Halogenated Flavonoid Derivatives Display Antiangiogenic Activity

**DOI:** 10.3390/molecules27154757

**Published:** 2022-07-25

**Authors:** Mai Khater, Kimberly A. Watson, Samuel Y. Boateng, Francesca Greco, Helen M. I. Osborn

**Affiliations:** 1 School of Pharmacy, University of Reading, Whiteknights, Reading RG6 6AD, UK; m.a.a.khater@pgr.reading.ac.uk; 2 Therapeutic Chemistry Department, Pharmaceutical & Drug Industries Research Division, National Research Centre, Cairo 12622, Egypt; 3 Institute of Cardiovascular and Metabolic Research, School of Biological Sciences, University of Reading, Whiteknights, Berkshire RG6 6AS, UK; k.a.watson@reading.ac.uk (K.A.W.); s.boateng@reading.ac.uk (S.Y.B.)

**Keywords:** angiogenesis, flavonoids, flavones, cancer, antiangiogenic, SAR

## Abstract

Antiangiogenic agents attenuate tumours’ growth and metastases and are therefore beneficial as an adjuvant or standalone cancer regimen. Drugs with dual antiproliferative and antiangiogenic activities can achieve anticancer efficacy and overcome acquired resistance. In this study, synthetic flavones (**5a**,**b**) with reported anticancer activity, and derivatives (**4b** and **6a**), exhibited significant inhibition of endothelial cell tube formation (40–55%, 12 h) at 1 µM, which is comparable to sunitinib (50% inhibition at 1 µM, 48 h). Flavones (**4b**, **5a**,**b** and **6a**) also showed 25–37% reduction in HUVECs migration at 10 µM. In a Western blotting assay, **5a** and **5b** subdued VEGFR2 phosphorylation by 37% and 57%, respectively, suggesting that VEGFR2 may be their main antiangiogenic target. **5b** displayed the best docking fit with VEGFR2 in an in silico study, followed by **5a**, emphasizing the importance of the 7-hydroxyl group accompanied by a 4−C=S for activity. Conversely, derivatives with a 4-carbonyl moiety fitted poorly into the target’s binding pocket, suggesting that their antiangiogenic activity depends on a different target. This study provides valuable insight into the Structure Activity Relationships (SAR) and modes of action of halogenated flavones with VEGFR2 and highlights their therapeutic potential as antiangiogenic/anticancer lead compounds.

## 1. Introduction

Vessel formation is essential for early organogenesis, and maintaining the normal body growth and function that follow [1]. Vasculogenesis is the earliest stage of vessel formation where the assembly of endothelial cells, differentiated from angioblasts, into primitive blood networks takes place. Angiogenesis, on the other hand, involves sprouting of pre-existing endothelial cells via proliferation, migration and tube formation to form more sophisticated vascular networks [2,3,4]. Dysregulation of vessel formation and growth is reflected in the pathologies of a daunting list of disorders dependant on either excessive angiogenesis, such as cancer, arthritis, psoriasis, endometriosis and obesity, or a lack of angiogenesis such as neurodegeneration, diabetes, hypertension, atherosclerosis and osteoporosis [1,4,5]. Under healthy conditions, angiogenesis is regulated and sustained by a vast and interlocked number of signalling proteins. The vascular endothelial growth factor (VEGF) family predominates this regulatory role [4,5,6] by stimulating vessel enlargement and branching through an interplay with several other protein families like platelet derived growth factor (PDGF), angiopoietins (ANG), fibroblast growth factor (FGF), hypoxia-inducible factors (HIFs) and matrix metalloproteinases (MMPs) [1,6,7]. A detailed description of the role of each family can be found in a review by Carmeliet, P. et al. [1]. 

Tumours rely heavily on an increased blood supply due to their aggravated need for oxygen and nutrients. In that context, angiogenesis is promoted during the early stages of tumour development and plays a key part in cancer metastases [8,9]. Tumour expression of angiogenesis promotor proteins like VEGF, interleukin-8 (IL-8) and HIF-1α [8] leads to the formation of a heterogeneous, leaky and highly branched tumour vasculature [10,11]. Hence, antiangiogenic therapy is gaining increased momentum as an integral part of the current clinical treatments for cancer. The anti-VEGF antibody bevacizumab (Avastin) and the multi-kinase inhibitors sorafenib (Nexavar) and sunitinib (Sutent) are examples of the antiangiogenic agents that are currently FDA approved for use in multiple malignancies’ treatment protocols [10,12,13]. Sunitinib, for instance, has shown notable efficacy in several clinical trials as a first-line treatment of patients with metastatic renal cell carcinoma (RCC) and second-line treatment for the treatment of inoperable metastatic gastrointestinal stromal tumours [14,15]. Additionally, sunitinib effectively extended tumour progression time in RCC and pancreatic neuroendocrine tumours by up to 8 months [15,16]. 

Despite the perceived success of antiangiogenic agents in prolonging progression-free survival rates among cancer patients, their impact on overall survival was limited [17]. The downfalls of the available antiangiogenic agents are often attributed to drug resistance [8,10,17]. Multiple strategies have been proposed to overcome these limitations. For instance, using drugs such as Paclitaxel that exploit both cytotoxic and antiangiogenic effects can help achieve high efficacy with an escape from drug resistance mechanisms resulting from vasculogenic mimicry [8,17,18]. Through this resistance mechanism, cancer cells can form endothelial like vascular assemblies by acquiring endothelial cell features [19,20]. Additionally, direct antiangiogenic agents are less likely to induce resistance than indirect ones. This is because direct inhibitors target proangiogenic factors or receptors located on endothelial cells, that are genetically stable, while the latter affects gene expression on unstable cancer cells [8,21]. Moreover, cancer cells can achieve drug resistance and maintain their vascular network by shifting their reliance on one proangiogenic factor to another [8]. Hence, affecting more than one target offers an alternative means by which drugs can bypass resistance. 

Flavonoids are one of the most extensively studied chemical classes of compounds for their general therapeutic effects owing to their abundance in nature and versatile pharmacological activities that include their anticancer, anti-inflammatory, neuroprotective and cardioprotective profiles [5,22]. There are several ongoing clinical trials on the effects of flavonoids on the mentioned indications but mostly as a dietary supplement [23]. With respect to their antiangiogenic activities, flavonoids are reported to inhibit numerous main targets such as vascular endothelial growth factor receptor 2 (VEGFR2) [12,24,25,26], epidermal growth factor receptor (EGFR) [27,28] and MMP-2, 9 [29,30,31,32], in addition to interfering with the relevant signalling pathways, namely, mitogen-activated protein kinase (MAPK/ERK) [27], phosphoinositide 3-kinase (PI3K) [5,7,33], HIF1-α/Akt [34,35] and IL-6/STAT3 [27]. 

Despite the abundance of literature on flavonoids, research efforts are challenged by the vast number of common dietary flavonoids and their countless therapeutic applications. Our recent studies concentrated on filling this gap by deciphering the key structural features required for the antiangiogenic activities of flavonoids. The first study focused on structural optimisation of the two common natural flavonoids, quercetin and luteolin [12], for antiangiogenic activity, and this was followed by our in depth meta-analysis of the antiangiogenic activities of a wide range of natural and synthetic flavonoids [5]. Both studies reported high antiangiogenic activities for flavonoids in general, and flavones in particular, thus directing our interest toward the future development of therapeutically active flavone leads. Therefore, in this study we sought to investigate the antiangiogenic potential of halogenated flavones (**5a**,**b**) that showed promising antiproliferative and growth inhibitory activities against several cancer cell lines (e.g., IC_50_ against breast cancer cell line (MCF-7) = 4.9 and 1 µM, respectively; NCI GI_50_ against MCF-7 = 1.46 µM and 0.18 µM, respectively) in our previous report [36]. The effects of dimethoxy and/or 4-oxo substitution on the in vitro antiangiogenic activity of the dihydroxyl 4-thioflavones (**5a**,**b**) were also examined in order to gain a better understanding of their structure activity relationships (SARs). These lead compounds could offer the benefit of having a dual antiangiogenic and/or anticancer activity, which addresses some issues faced by the currently available antiangiogenic agents. 

In summary, the direct in vitro antiangiogenic activities of a synthesized set of halogenated flavones were evaluated against endothelial cells’ VEGF-mediated tube formation. In order to evaluate how inhibition of the overall tube formation transcends into suppression of the key steps involved in the process of angiogenesis, candidates showing the highest tube inhibition activities were assessed for their abilities to withhold VEGF-stimulated endothelial cell migration. In that context, interference with the VEGF/VEGFR2 pathway, as a predominant regulator of angiogenesis, was explored for this panel of compounds via Western blotting. A SAR study then determined the main structural features required to inhibit VEGFR2 phosphorylation for the tested set of flavones, and a comparison was made between the collected data to establish connections between the observed trends of activity using the different assays. Finally, molecular docking studies were carried out, in silico, to investigate whether the mode of interaction of each compound with VEGFR2 explained the biological activity observed.

## 2. Results and Discussion

### 2.1. Synthesis

**Scheme 1:**
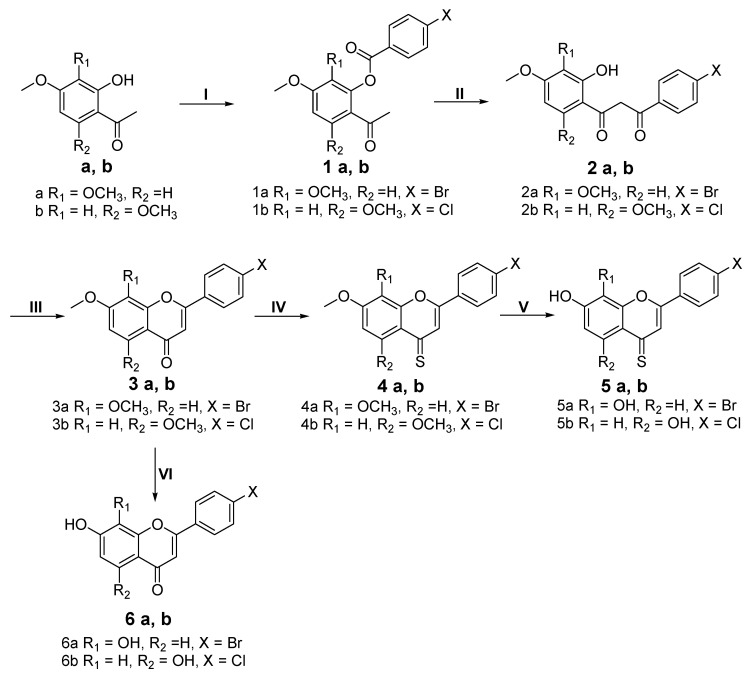
Synthesis of oxo and thio-p-halo-phenyl flavone derivatives. (I) Halobenzoyl Chloride, DBU, pyridine, 75 °C, 2 h; (II) Pyridine, KOH, 50 °C, 2 h; (III) Glacial acetic acid, 1% H_2_SO_4_, 90–110 °C, 1 h; (IV) Dry toluene, Lawesson’s reagent, 110 °C, 4 h; (V, VI) Dry DCM, BBr_3_, room temp, 4 h.

Two series of 4′-bromo (series a) or 4′-chloro (series b) flavones were synthesized according to Scheme 1, following the reported method [36], to produce compounds **1a**,**b**–**6a**,**b**. The structures of the synthesized compounds were confirmed using ^1^H and ^13^C NMR spectroscopic analysis, mass spectrometry and IR spectroscopy, and they matched the reported data. The first step involved an esterification reaction of 2-hydroxy, 3,4 or 4,6-dimethoxy acetophenone and bromo- or chlorobenzoylchloride, respectively. Double-doublet signals for the phenyl halide rings appeared in the ^1^H NMR spectra of **1a**,**b** at δ 8.03 (*J* = 8.0 Hz) and 7.75 (*J* = 8.0 Hz) ppm, respectively, indicating successful esterification. The resulting esters (**1a**,**b**) underwent a Baker-Venkataraman’s rearrangement in the presence of KOH, affording the diketone intermediates (**2a**,**b**) in 87 and 80% yields, respectively. This was followed by acid assisted cyclic dehydration to afford the methoxy flavones (**3a**,**b**) in 88 and 77% yields, respectively. Cyclisation was evident from the presence of the olefinic –CH proton at δ 6.75 and 6.64 ppm for **3a**,**b**, respectively, in their ^1^H NMR spectra. The 4-thio derivatives (**4a**,**b**) were obtained via a thionation reaction of the 4-C=O parents (**3a**,**b**) using Lawesson’s reagent in 88% and 77% yields, respectively. Thionation resulted in around 25 ppm downfield shift of the carbon at position 4 in the ^13^C NMR spectra of **4a**,**b**. Subsequent demethylation of both the 4-oxo and 4-thio methoxy flavones (**3a**,**b** and **4a**,**b**) was carried out using BBr_3_ in anhydrous DCM affording the hydroxyl derivatives (**5a**,**b** and **6a**,**b**) in favourable yields (60–97%), where the two OCH_3_ peaks disappeared from their ^1^H and ^13^C NMR spectra. The broad OH peaks also appeared in the IR spectra of **5a**,**b** and **6a**,**b** at ν = 3501, 3358, 3365 and 3350 cm^−1^, respectively. Purities of the synthesized compounds were >90%, as determined by HPLC. Structural elucidation from the spectral data can be found in full in the Supporting Information File. 

### 2.2. Cytotoxicity on HUVEC Cells 

**Figure 1:**
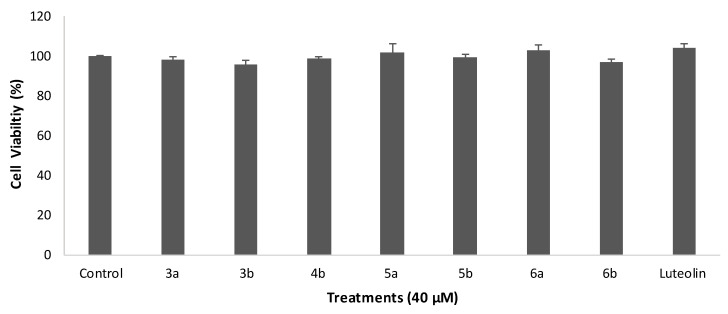
Cell viability of HUVECs at 40 µM of tested flavones. Data are expressed as mean ± standard error of the mean (SEM), *n* = 3.

The trypan blue exclusion assay was applied to the tested flavones with HUVEC cells to ensure their biocompatibilities and determine that the observed antiangiogenic effects are not a consequence of cytotoxicity. Treatment with a concentration four times higher (i.e., 40 µM) than the highest concentration used for the antiangiogenic evaluation studies (i.e., 10 µM) for 24 h was chosen for this cytotoxic assay. As shown in Figure 1, all of the tested compounds retained ~100% viability of the cells with no observed cytotoxic activities (no statistically significant difference compared to control in all cases, *p* > 0.05). 

### 2.3. In Vitro Tube Formation Assay

**Figure 2:**
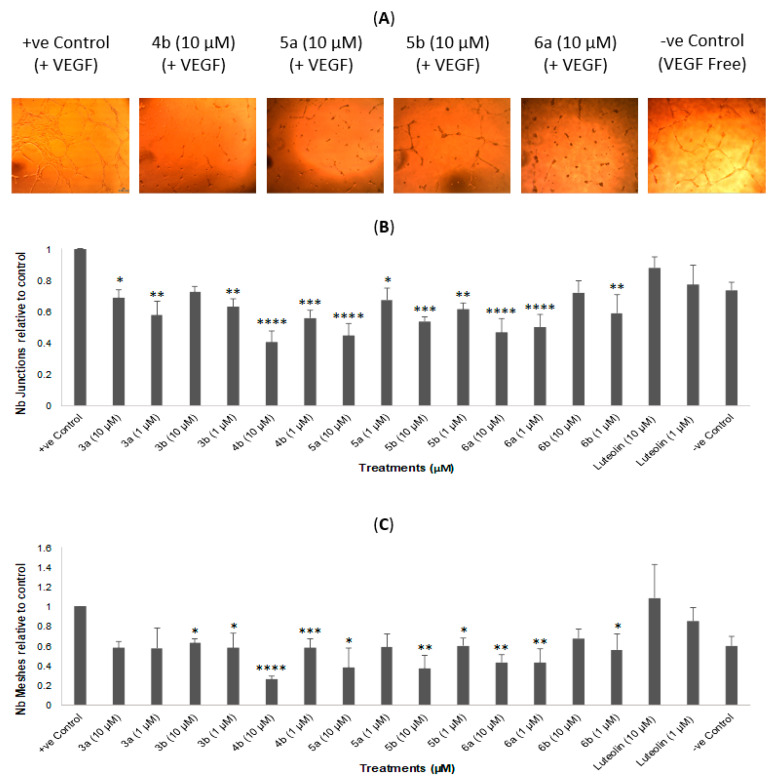

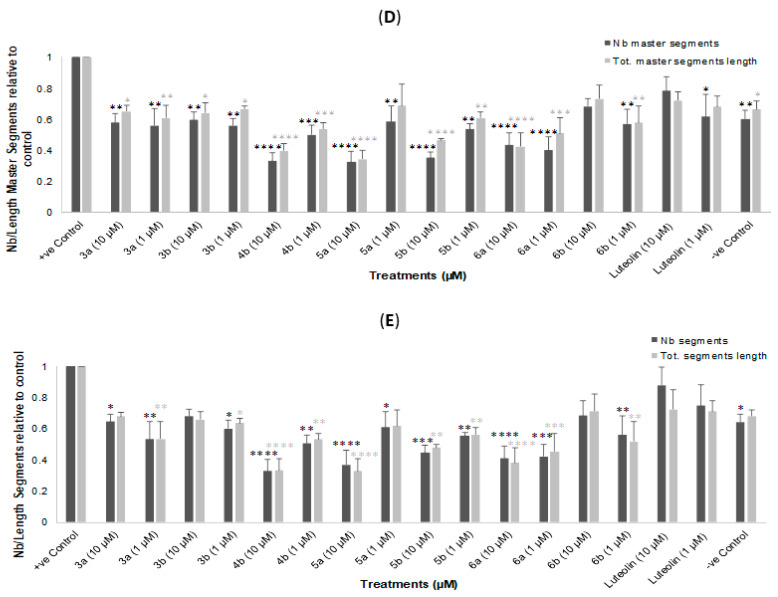
Antiangiogenic activity of flavonoid derivatives on in vitro HUVEC tube formation after 12 h expressed as a ratio to the +ve control (10 ng/mL VEGF-enriched media). (**A**) Representative images of tube formation assay at 4× magnification. Images were analysed using Angiogenesis Analyzer macro in ImageJ software; (**B**) number of junctions, (**C**) number of meshes, (**D**) number and length of master segments and (**E**) number and length of segments. Data are expressed as mean ± standard error of the mean (SEM), *n* = 3. Statistical significance was estimated with respect to the +ve control by one-way ANOVA, followed by Dunnett’s multiple comparison test (* *p* < 0.05, ** *p* < 0.01, *** *p* < 0.001, **** *p* < 0.0001).

The antiangiogenic activities of the synthesised panel of flavones were evaluated in vitro using the Matrigel tube formation assay with the exception of compound **4a** due to solubility issues for this compound. This assay offers a practical method to evaluate the overall antiangiogenic effects of potential leads as it covers the fundamental steps involved in the process of angiogenesis [37]. Herein, two series of synthetic flavones **a** and **b** in addition to luteolin (common natural flavone with in vitro and in vivo antiangiogenic activity [38]) as a reference standard, were screened for their ability to inhibit tube formation of HUVEC cells after 12 h at 10 µM and 1 µM concentrations. As the aim was to develop potent antiangiogenic flavone leads, test concentrations were selected in the low concentration range, since high concentrations of antiangiogenic drugs are often linked to increased toxicity in vivo [39]. The parameters measured in this assay via the Angiogenesis Analyser plugin [40]; the number (nb) of junctions and meshes, number and length of master segments and segments offer high sensitivity in the detection of different features of angiogenesis [41]. As shown in Figure 2, the reference standard luteolin showed around 30% decrease in tube formation, in agreement with previous reports [38,42]. In this regard, all of the evaluated derivatives demonstrated higher inhibition of tube formation than the active reference luteolin (Figure 2). 

Out of the seven tested flavones, compounds **4b**, **5a**,**b** and **6a** showed the highest antiangiogenic activities against the measured elements of angiogenesis. Compound **4b** exhibited the strongest antiangiogenic activity at 10 µM with 60% inhibition of the number of junctions and total master segments length (*p* < 0.0001); 70% inhibition of number of master segments, number and total segments length (*p* < 0.0001); and 75% inhibition of number of meshes (*p* < 0.0001). The two 4-thio di-hydroxy derivatives (**5a**,**b**) also showed strong reduction in tubule formation, whereas the 7,8-dihydroxy 4′-bromo derivative (**5a**) showed slightly better activity (64% inhibition) than the 5,7-dihydroxy 4′-chloro analogue (**5b**) that resulted in 60% tube formation inhibition. At 1 µM concentration, derivative **6a** showed the strongest activity (55% inhibition, *p* < 0.0001) among all tested compounds, which is comparable to the clinically used anticancer/antiangiogenic drug sunitinib (50% inhibition after 48 h) at the same concentration [43]. At 10 µM, compound **6a** followed the trend of the other three mostly active compounds with 57% inhibition of all the measured tube formation elements. 

A pattern of the lower 1 µM concentration, demonstrating comparable tube formation inhibition activity to the 10 µM concentration, was observed with the flavone derivatives bearing a C=O group at position number 4 (**3a**,**b** and **6a**,**b**). The observed increase in activity ranged from ~2% to 14% at best. The 1 µM of compound **3a,** for example, exhibited 43% inhibition compared to 36% inhibition at 10 µM, while derivative **6b** showed 44% inhibition at 1 µM versus 30% at 10 µM. This finding suggests that 4-C=O flavones saturate their target protein and thus reach maximum reactivity at a concentration lower than 10 µM. Interestingly, the same observation was not reiterated with 4-C=S derivatives (**4b** and **5a**,**b**), which might indicate they exert their antiangiogenic activity via a different mechanism of action. In general, the 4-thio derivatives exhibited higher antiangiogenic activity than their 4-oxo counterparts, further supporting the hypothesis that these act via a different mode of action, which was investigated in the following assays. The increase in antiangiogenic activity due to thionation was much more significant in series b (30% difference in inhibition, *p* < 0.0001) than in series a (7% difference in inhibition, *p* < 0.05) (Table 1). This can be attributed to the antiangiogenic activity of series a being already high (e.g., **6a** inhibited tube formation by 57%) before thionation, which gives little room for improvement upon the 4-oxo-thio substitution. 

To gain more insight into the possible mechanisms of action of these flavones and their activity pattern, compounds showing more than 50% overall tube formation inhibition at 10 µM (**4b**, **5a**,**b** and **6a**) were selected for further antiangiogenic evaluation. 

### 2.4. Wound Healing (Scratch) Assay

**Figure 3:**
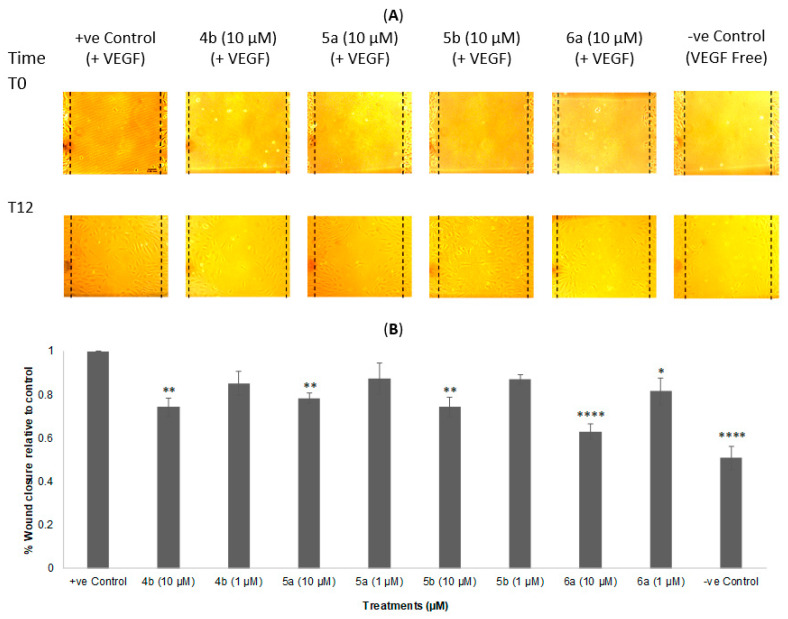
In vitro HUVEC wound closure (migration) inhibition activity of flavonoid derivatives (**4b**, **5a**,**b** and **6a**) expressed as a ratio to the +ve control (10 ng/mL VEGF-enriched media). (**A**) Representative images of scratch assay at 0 h and 12 h at 10× magnification. Images were analysed using ImageJ software; (**B**) % wound closure after 12 h as a ratio to +ve control. Data are expressed as mean ± standard error of the mean (SEM), *n* = 3. Statistical significance was estimated with respect to the +ve control by one-way ANOVA, followed by Dunnett’s multiple comparison test (* *p* < 0.05, ** *p* < 0.01, **** *p* < 0.0001).

A wound healing (scratch) assay was used to assess the effect of the most active flavones (**4b**, **5a**,**b** and **6a**) on the collective cell migration of HUVECs. As seen in Figure 3, the 4-thio derivatives (**4b** and **5a**,**b**) showed analogous inhibition of HUVECs’ VEGF mediated migration of around 25% (*p* < 0.01) at the 10 µM concentration and 15% at the 1 µM concentration after 12 h. Despite the 4-C=S derivatives being more active in the tubule formation inhibition assay, herein, compound **6a** (4-C=O flavone) resulted in the highest level of migration inhibition at both the 10 µM and 1 µM concentrations (37%, *p* < 0.0001 and 20%, *p* < 0.05, respectively). A previous SAR study evaluating the antiangiogenic activity of a panel of flavonoids similarly reported a rise in migration inhibition activity upon substitution of the 4-C=S with a 4-C=O moiety [12]. This reinforces the previous observations derived from the tube formation assay that the 4-thio and 4-oxo derivatives have different antiangiogenic mechanisms of action.

### 2.5. Western Blotting

**Figure 4:**
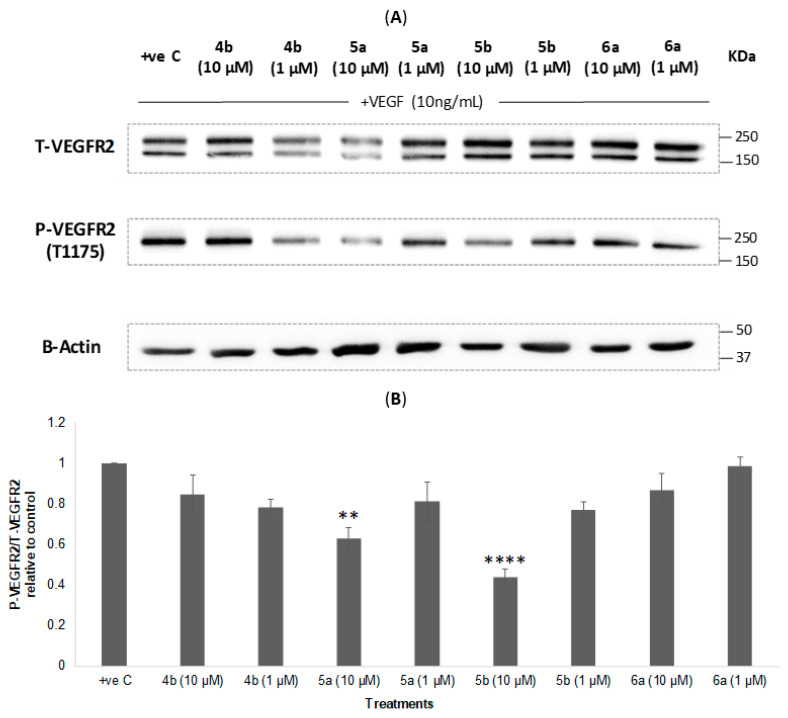
VEGFR2 induced phosphorylation inhibition activity of flavonoid derivatives (**4b**, **5a**,**b** and **6a**) on HUVEC cell lysates. (**A**) Representative Western blot images of the HUVECs proteins; T-VEGFR2, P-VEGFR2 and β-Actin. Images were analysed using ImageJ software; (**B**) VEGFR2-induced phosphorylation inhibition expressed as a ratio to the +ve control (10 ng/mL VEGF-enriched media). Data are expressed as mean ± standard error of the mean (SEM), *n* = 3. Statistical significance was estimated with respect to the +ve control by one-way ANOVA, followed by Dunnett’s multiple comparison test (** *p* < 0.01, **** *p* < 0.0001).

**Figure 5:**
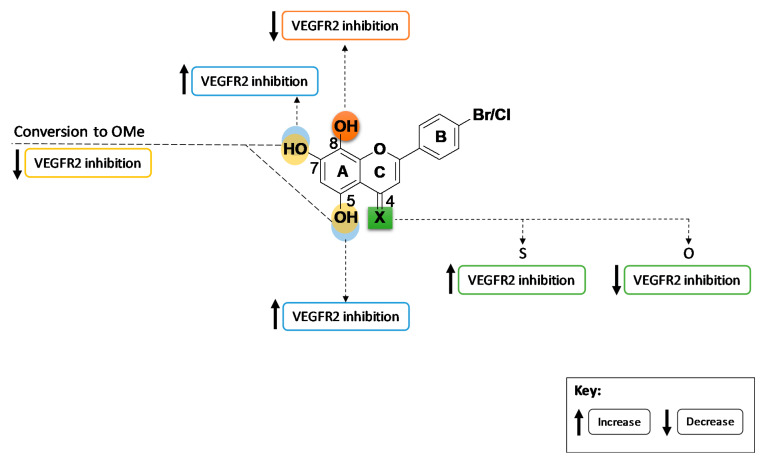
Summary of SAR of the tested panel of flavones on VEGFR2 phosphorylation inhibition.

VEGFR2 possesses six tyrosine phosphorylation sites that activate several proangiogenic downstream signalling cascades upon VEGF binding to its extracellular domain [44]. Since the flavone derivatives (**4b**, **5a**,**b** and **6a**) demonstrated promising VEGF-mediated tube formation and migration inhibition on endothelial cells, the next step was to test their abilities to inhibit VEGFR2 activation via interfering with TYR1175 auto-phosphorylation. TYR1175 phosphorylation is critical for the activation of VEGFR2 and its mediated signalling cascades that are responsible for endothelial cells survival, migration and permeability [12,44,45]. As shown in Figure 4, the thio-flavone **5b** significantly diminished VEGFR2 phosphorylation by 57% (*p* < 0.0001) at 10 µM compared to the positive control. Its series a counterpart (**5a**) showed less but statistically significant VEGFR2 phosphorylation inhibition of 37% (*p* < 0.01), also at the 10 µM concentration. Interestingly, both derivatives **4b** and **6a** did not follow a similar trend with a minor and insignificant inhibition of 15%. Looking into the structures of the tested compounds, it becomes clear that the two active compounds (**5a**,**b**) share two structural features, which are a 4-thio substitution combined with di-hydroxyl groups. Furthermore, the 5,7-dihydroxyl position appears to be more favourable for VEGFR2 phosphorylation inhibition than the 7,8-disubstitution. In general, a dihydroxyl substitution at positions 5 and 7 was designated to be important for a wide range of the reported flavonoids’ pharmacological activities [5,46,47,48,49]. A summary of the structural VEGFR2 inhibitory activity relationship is shown in Figure 5. 

**Figure 6:**
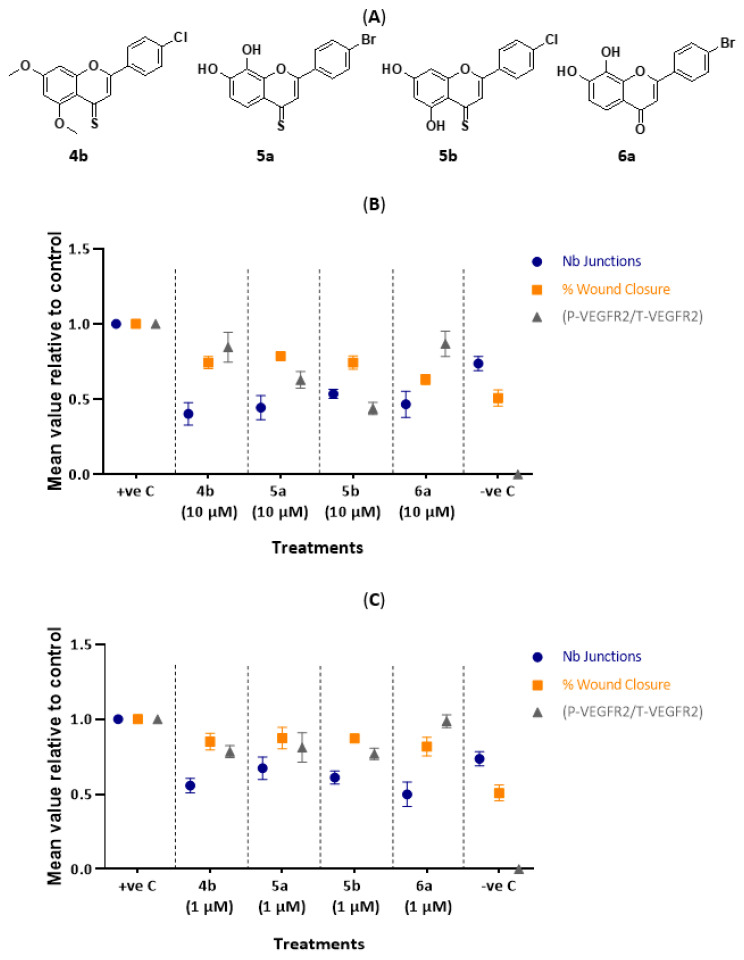
Comparison between tube formation (represented by nb junctions), scratch and Western blotting assay results of flavonoid derivatives (**4b**, **5a**,**b** and **6a**). (**A**) Chemical structures of **4a**, **5a**,**b** and **6a**; (**B**) comparison of 10 µM concentration; (**C**) comparison of 1 µM concentration.

Figure 6 features the three sets of results obtained from the tube formation, migration and VEGFR2 phosphorylation assays for each of the four compounds, in comparison to each other. This comparison can help to understand how the inhibition of VEGFR2 activation translates into the antiangiogenic activities seen for compounds **4b**, **5a**,**b** and **6a** in the tube formation and migration assays. In general, it is noted that the tube formation activities were higher than the other measured activities indicating that the observed collective arrest of tubulogenesis resulted from interaction with one or more different targets. The VEGFR2 phosphorylation and tube formation activities of the 4-C=S, di-hydroxy flavones (**5a**,**b**) though were in good agreement, indicating that VEGFR2 may be the main target by which these derivatives exert their antiangiogenic activities. No statistically significant differences were found between the extent of VEGFR2 and migration inhibition for this set with the exception of **5b** at 10 µM. Since compounds **4b** and **6a** did not show a noticeable activity on VEGFR2, no correlation was observed with their tube formation inhibitory activity; however, wound closure results mirrored the VEGFR2 activity for the di-methoxy derivative, **4b**. This suggests that the slight activity observed for **4b** on inhibition of VEGFR2 phosphorylation manifested itself on the migration of HUVECs while the high decline in tube formation is a contribution from another target. This phenomenon termed polypharmacology [5] has been reported for flavonoids as they can interfere with one or more targets to achieve a certain effect [50,51,52]. As mentioned earlier, flavonoids can interact with numerous angiogenic targets such as EGFR [27,28], VEGFR2 [12,24,25,26] and MMPs [29,30,31,32], as well as the MAPK/ERK [27], HIF1-α/Akt [34,35] and IL-6/STAT3 [27] pathways. Regarding the 4-C=O flavone, **6a**, the parallel trends observed for tube formation and migration inhibition activities was not a consequence of any VEGFR2 interaction. 

### 2.6. Molecular Docking

**Figure 7:**
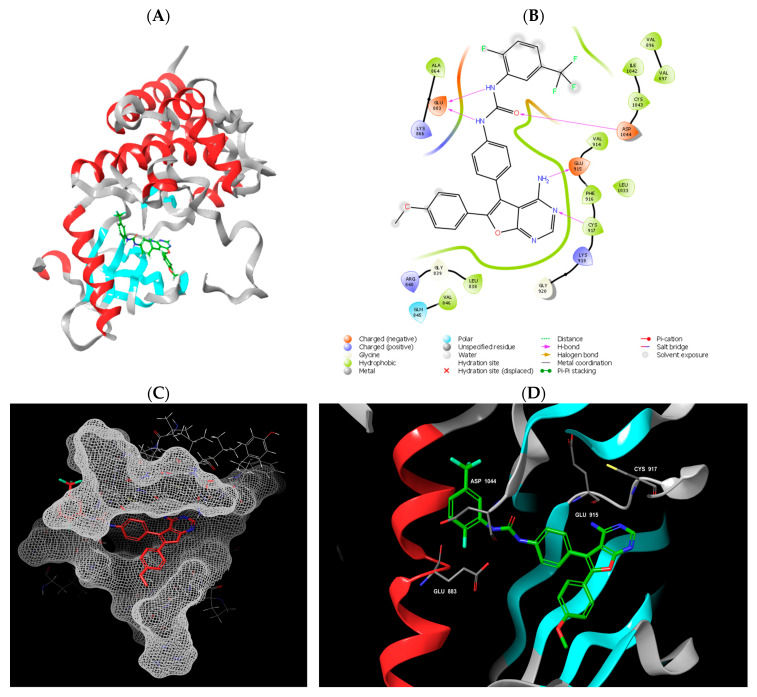
Docking of the original co-crystallized ligand (N-{4-[4-amino-6-(4-methoxyphenyl)furo[2,3-d]pyrimidin-5-yl]phenyl}-n’-[2-fluoro-5-(trifluoromethyl)phenyl]urea) with VEGFR2 (1YWN). (**A**) Ribbon representation showing ligand pose in chain A; (**B**) 2D interaction of ligand with binding pocket; (**C**) mesh representation of binding pocket surface interaction with ligand; (**D**) 3D interaction of ligand with binding pocket showing involved amino acids.

The interaction of compounds with the highest antiangiogenic activity (**4b**, **5a**,**b** and **6a**) with the VEGFR2 target protein (X-ray crystal structure, PDBid: 1YWN) was studied in silico. The docking study provided valuable insight about how these flavones interact with VEGFR2, at a molecular level, and explained the order of activity of VEGFR2 phosphorylation inhibition (**5b** > **5a** > **4b** > **6a**), as observed in the Western blotting assay. The remaining synthesized derivatives (**3a**,**b** and **6b**) also were included in the molecular docking with VEGFR2 to further elucidate the SAR of this particular panel of halogenated flavones. In order to validate the docking procedure, the original ligand (4-amino-furo [2,3-d]pyrimidine) co-crystallized with VEGFR2 was removed from the receptor and re-docked with the prepared active site. Superimposition of both the original and re-docked ligand resulted in a Root Mean Square Deviation (RMSD) of 0.97 Å, which is well within the 2 Å grid resolution used for docking [53]. Moreover, the binding mode of the docked ligand was compared with the co-crystallized structure solution (PDBid: 1YWN) [54] and demonstrated the same interactions, as shown in Figure 7. 

**Figure 8:**
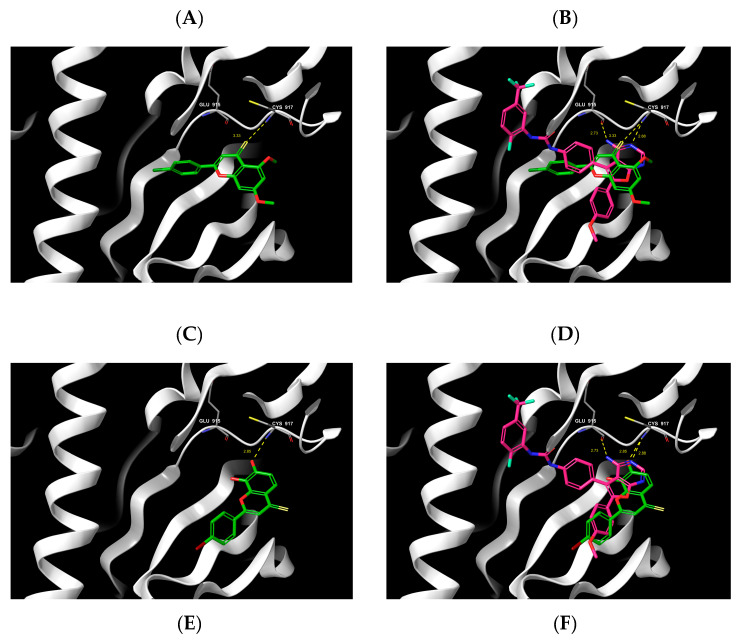

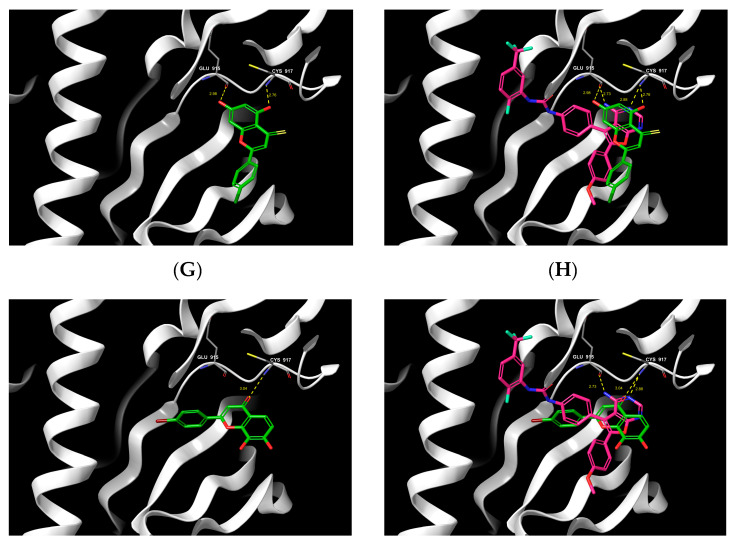
3D interaction of flavonoid derivatives (**4b**, **5a**,**b** and **6a**) (green) with the ATP-binding pocket of VEGFR2 and compared to co-crystallized ligand (pink). (**A**,**B**) Flavonoid **4b**; (**C**,**D**) **5a**; (**E**,**F**) **5b**; (**G**,**H**) **6a**.

**Figure 9:**
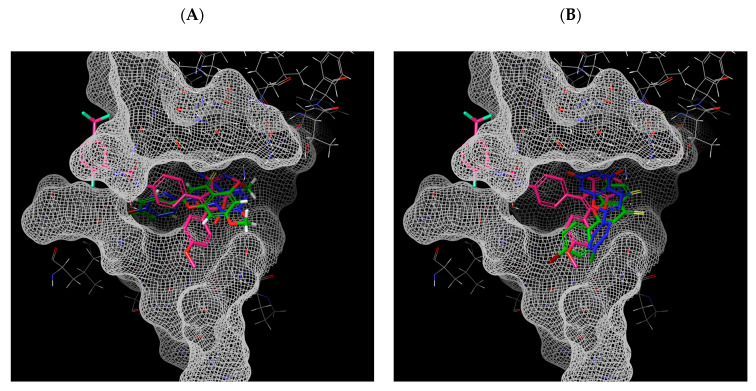
Mesh representation of flavonoid derivatives (**4b**, **5a**,**b** and **6a**) in the ATP-binding pocket of VEGFR2 compared to co-crystallized ligand (pink). (**A**) Less active horizontal orientation of flavonoids **4b** (green) and **6a** (blue); (**B**) more active vertical orientation of flavonoids **5a** (green) and **5b** (blue).

The VEGFR2 binding pocket consists of three main regions: an ATP-binding domain which contains Glu915 and Cys917; a DFG domain that controls the receptors’ active/inactive conformations and contains GLu883 and Asp1044 residues in addition to the allosteric hydrophobic region containing residues like Val896, Cys1043, etc. [55,56]. A flat heteroaromatic ring system occupying the ATP-binding region and possessing at least one nitrogen atom, such as the aminopyrimidine moiety of the co-crystallized ligand, is a core structural feature among the majority of VEGFR2 inhibitors [55]. Flavonoids fit well with these criteria owing to their planar heteroaromatic ring structures. The majority of compound **5b**’s conformations adopted a pose in which ring A is closely stacked on the ligand’s aminopyrimidine ring, thus mimicking its binding mode inside the ATP-binding cavity of VEGFR2. Figure 8E,F shows how the OH groups at positions 5 and 7, of the top docked pose of **5b**, acted as bioisosteres for N and NH_2_ of the ligand forming hydrogen bonds (HB) with Cys917-N (2.76 Å) and Glu915-O (2.98 Å), respectively. This orientation explains the high activity that **5b** showed on VEGFR2 phosphorylation inhibition. As for the less active thioflavone **5a**, slightly more than half the 20 generated conformations adopted a favourable vertical alignment, parallel to that of **5b**. **5a**’s top conformer was capable of forming one HB with Cys917-N (2.85 Å) via the O at position 7 as shown in Figure 9B. On the other hand, the loss of VEGFR2 inhibitory activity witnessed for derivatives **4b** and **6a** can be explained in light of their turned (horizontal) alignment inside the ATP-binding domain (Figure 9A). Unlike the active derivatives **5a**,**b**, compounds **4b** and **6a** formed HB with the important residue Cys917-N but with their 4-C=S (3.33 Å) and 4-C=O (3.04 Å) moieties, respectively, instead of the O in their OH groups (Figure 8A,B,G,H).

**Figure 10:**
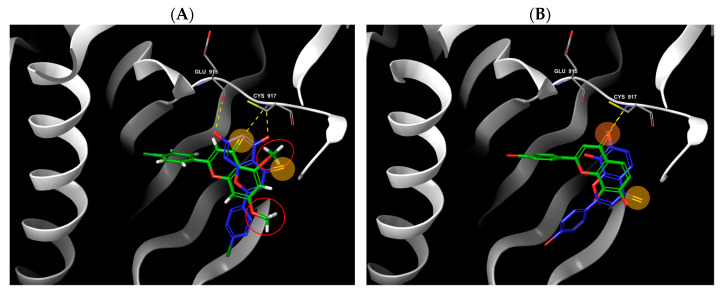
Cartoon representation showing the different orientations (horizontal or vertical) of flavonoid derivatives (**4b**, **5a**,**b** and **6a**) in ATP-binding pocket of VEGFR2 resulting from the highlighted structural differences (yellow shadow, C=S; red circle, OCH_3_; red shadow, C=O). (**A**) 4b (green, horizontal orientation) vs. **5b** (blue, vertical orientation); (**B**) **5a** (blue, vertical orientation) vs. 6a (green, horizontal orientation).

Figure 10A illustrates how the substitution of OH in **5b** with OCH_3_ groups in **4b** leads to a different binding mode in which the C=S is directed to the inside of the cavity rather than the solvent exposures surface as seen in **5b**. The bulkiness and branching of the OCH_3_ groups hindered their ability to occupy the desirable binding position where they can face the two important residues Glu915 and Cys917. A comparison between the active 4-thioflavone, **5a** and its 4-oxo derivative **6a** is also highlighted in Figure 10B. Here, **6a** adopted a horizontal alignment, where the carbonyl group was directed towards the Cys917 residue possibly due to the better hydrogen bond acceptor (HBA) character of the carbonyl O over the hydroxyl O. Thus, molecule **6a** has a higher number of conformations in which the C=O is facing the inside of VEGFR2 binding cavity where it can interact with several amino acids via HB. To further investigate the effect of 4-C=O functionality on VEGFR2, the binding modes of compounds (**3a, b** and **6b**) were examined. Indeed, none of the conformations adopted by flavones (**3a**,**b** and **6b**) showed the distinctive binding mode of the ATP-binding scaffold of the ligand or the active compound **5b**, with the exception of 3 out of 20 conformations for compound **6b**. Comparison between all of the docked poses of each flavone inside the binding groove (Supporting Information Figure S10) conveyed the same message. That is, most of compound **5b**’s conformations are a close match to the ligand’s ATP-binding scaffold, followed by the less active derivative **5a**, whereas the rest of the conformations of the studied flavones have shown little similarity to that particular active mode of binding. 

## 3. Materials and Methods

Cell Culture: HUVECs were purchased from Sigma-Aldrich (ECACC) and cultured in EGM-2 (EBM with SingleQuotesTM kit: foetal bovine serum (FPS), fibroblast growth factor B, epidermal growth factor, vascular endothelial growth factor (VEGF), insulin-like growth factor-1, heparin, hydrocortisone) (Lonza, Belgium). The cells were incubated at 37 °C and 5% CO_2_. HUVEC cells were at passage 3–5 when used in the experiment and were not further sub-cultured. Bovine serum albumin (BSA) was purchased from Sigma Aldrich, Dorset, UK. Recombinant human VEGFR-A165 was purchased from Peprotech, UK. The primary antibodies directed against phosphorylated tyrosine-1175 site in the VEGFR2 (KDR), total VEGFR2, actin and Horseradish peroxidase-conjugated secondary antibody were purchased from cell signalling, UK. Enhanced chemiluminescence (ECL) detection solutions were purchased from Bio-Rad, Watford, UK. The 0.45 µm PVDF membrane was purchased from Thermo Fisher Scientific, UK. Reagents for phosphate-buffered saline (PBS) for cell culture were purchased from Sigma Aldrich, Gillingham, UK. PBS (pH 7.4) was freshly prepared in lab and solution pH was checked before used. Images were captured using 1.3 M microscope digital eyepiece camera. ImageJ software was used to quantify tube formation, cell migration and Western blot band density. The stock solutions of the test compounds (20 mM) were prepared in 100% sterile DMSO. These stocks were then appropriately diluted with the complete culture medium, and DMSO levels were maintained below 0.1% in the test concentrations. Compound **4a** was not tested due to insolubility in any of the organic solvents suitable for biological evaluations. 

Statistical analysis: Statistical analysis was carried out against the control group by one-way ANOVA followed by Dunnett’s post hoc test using GraphPad Prism 6. Statistical significance value was set at *p* < 0.05.

### 3.1. Synthesis

Synthesis in this study followed the previously published protocol [36]. Structural characterisation of the synthesized derivatives can be found in the Supporting Information file.

### 3.2. Biological Assays

#### 3.2.1. Cytotoxicity on HUVECs

Cytotoxic activity of treatments on HUVECs using trypan blue exclusion assay [57]. HUVECs were seeded in 96-well plates at 1 × 10^5^ cells/mL and cultured for 24 h. Cells were then treated with either luteolin, the synthesised derivatives at 40 µM or culture medium (control) for 24 h. After 24 h, solutions were removed, and cells were washed with 100 µL PBS followed by the addition of 50 µL trypsin-EDTA and incubation for 5 min to detach the cells. Then, 50 µL of EGM-2 media was added to the wells. Next, 50 µL aliquots of the cell suspension were mixed with equal volume of trypan blue (TB) 0.2% *v*/*v* (prepared from 0.4% TB diluted with PBS), and then the cells were counted using a haemocytometer. The number of viable and dead cells were counted manually and % cell viability of each treatment was expressed as % of control using the equation:

% Cell Viability = (Cell viability in treatment/Cell viability in control) × 100

where Cell Viability was calculated as follows:

Cell Viability = Number of dead cells/Total number of cells (viable and dead)

#### 3.2.2. Endothelial Cell Tube Formation Assay

HUVECs were cultured until confluency and then serum starved (0.1% serum) for 24 h. The tube formation assay followed the reported method [58]. Briefly, 96-well plates were coated with 50 µL of Corning™ Matrigel™ GFR Membrane Matrix (Fischer Scientific, Loughborough, UK) at 4 °C and incubated at 37 °C for 1–2 h to solidify. Serum-starved HUVECs were seeded on the solidified Matrigel at 3 × 10^5^ cells/mL and treated with medium containing VEGF (10 ng/mL) and either luteolin or one of the synthesised derivatives (at 10 µM or 1 µM). Plates were incubated for 12 h and photos covering the whole well area were taken using 4× magnification power of an inverted light microscope. Number of junctions, number of meshes, number and length of segments and master segments were quantified from the taken Images via the Angiogenesis Analyzer plugin [40] in ImageJ software [59]. The Angiogenesis Analyzer plugin proved to be an efficient tool in characterizing the branching of endothelial cells into tube networks as well as identifying various elements of endothelial tube formation [40]. Data were represented as a ratio to the positive control (VEGF enriched).

#### 3.2.3. Scratch Assay

Following the reported method [12], HUVECs were seeded in 12-well plates at 3 × 10^4^ cells/well and cultured until 80–90% confluency. Afterwards, they were serum starved (0.1% serum) for 24 h to inactivate the cell proliferation. A scratch was performed on the cell monolayers using a 200 µL pipette tip. Cells were then washed twice with PBS and treated with medium containing VEGF (10 ng/mL) and either **4b**, **5a**,**b** or **6a** (at 10 µM or 1 µM). Images of the scratches were taken immediately after performing the scratch (*t* = 0 h) and at 12 h (*t* = 12 h). The area not covered by the cells was quantified using ImageJ software. The % of wound closure was calculated using the following equation:

100 × (Area of scratch at t0 − Area of scratch at t12/Area of scratch at t0)

#### 3.2.4. Western Blotting 

HUVECs were cultured in 6-well plates at 1 × 10^5^ cells/well and cultured for 48 h. After 48 h, they were serum starved (0.1% serum) for 24 h to inhibit cell proliferation. Cells were then treated with complete medium or compounds **4b**, **5a**,**b** or **6a** (at 10 µM or 1 µM) for 1 h followed by 30 min incubation with VEGF (10 ng/mL) to activate VEGFR2 phosphorylation. Afterwards, cells were washed twice with ice cold PBS and lysed using 100 µL radioimmunoprecipitation assay (RIPA) lysis buffer with 1% protease inhibitor and phosphatase inhibitor. The amount of protein present in the cell lysate was assessed using the DC (Bio-Rad) protein assay developed from Lowry’s protocol for protein assessment [60]. Protein lysates (15 µg) were separated by SDS-PAGE and then transferred to PVDF membranes. Membranes were blocked with 5% BSA in TBS-T buffer and then probed for P^Tyr−1175-^VEGFR2 (1:1000) and actin (1:1000). Protein bands were detected by incubating with horseradish peroxidase-conjugated secondary antibodies (1:5000) and visualised with enhanced chemiluminescence reagent using ImageQuant LAS 4000 (GE Healthcare, Hatfield, UK). Next, P^Tyr−1175-^VEGFR2 membranes were stripped from bound antibodies following Abcam protocol [61] and then re-probed for total VEGFR2 (1:1000). The density of bands was measured using ImageJ software. P-VEGFR2 was normalized to T-VEGFR2 and data were represented as a ratio to the positive control (VEGF enriched). 

#### 3.2.5. Molecular Docking

Molecular docking studies were carried out using the programme Surflex-Dock (SFXC) [62], as provided by Sybyl-X 2.1, according to the reported procedure [12]. The X-ray crystallographic structure of VEGFR2 in complex with a novel 4-amino-furo[2,3-d]pyrimidine was obtained from the Protein Data Bank (PDBid: 1YWN, 1.71 Å resolution) [54]. First, the docking procedure was validated by re-docking of the original pyrimidine ligand (extracted from the coordinate files, taken from PDBid: 1YWN) adding it into the prepared protein structure (details below). Superimposition of the ligand conformation resulting from docking and the original ligand co-crystallized with the protein structure, resulted in a RMSD value of 0.97 Å calculated by Maestro program V13.1 [63]. This RMSD value and the reproducible ligand–protein contacts indicated that the docking procedure is valid and reliable as are the results, which are well within the 2 Å grid spacing used in the docking procedure [53]. 

Docking studies for the new compounds of interest were performed using the program Surflex-Dock (SFXC) as provided by Sybyl-X 2.1. 

The protein structure was prepared for docking using the Biopolymer Structure Preparation Tool with the implemented default settings provided in the SYBYL programme suite. Hydrogens were added to the protein structure in idealised geometries, and an overall energy minimisation of the protein was performed using the MMFF94 force field, employing a conjugate gradient algorithm [64] with a convergence criterion of 0.5 kcal/mol A^−1^ and up to 5000 iterations. Finally, before the docking run, all water molecules, except molecule number 163 (previously shown to be involved in water-mediated ligand–protein interactions) were removed, and the ligand 4- amino-furo[2,3-d]pyrimidine was extracted from the coordinate file of the VEGFR2 receptor (1YWN). The protomol, representing the ligand-binding groove, was generated using a ligand-directed method, which allows for the docking of ligands into predefined sites, as defined by occupancy of any co-crystallised ligand at the site of interest. The Surflex-X docking algorithm docks a given ligand to a receptor using a flexible ligand and a semi-flexible receptor algorithm; in this case, the compounds were allowed to be fully flexible while the receptor was semi-flexible. This allows for the optimisation of potentially favourable molecular interactions, such as those defined by hydrogen bond and van der Waal forces. The docking results yield a docking score, which takes into consideration entropic, polar, hydrophobic, repulsive and desolvation factors. The docking scores are expressed in −log10 (K_d_) units to represent binding affinities, where K_d_ is the dissociation constant. The free energy of binding of the ligand to the protein was extrapolated from the equation:

Free energy of binding = RTlog_e_K_d_

Visualisation and analyses of the docking results as well as the molecular interactions of the docked ligands was performed using the program Maestro [63]. Potential hydrogen bonds were assigned if the distance between two electronegative atoms was less than 3.5 Å, whereas any separation greater than 3.5 Å, but less than 4.5 Å, was considered a van der Waal interaction.

## 4. Conclusions

In this study, the antiangiogenic potential of two series of structurally similar halogenated flavone derivatives (**3a**,**b**–**6a**,**b**) was evaluated, in vitro. Analysis of the findings of the different assays used suggested that structural modifications among the tested panel impacted their antiangiogenic behaviour and mode of action. While compounds with a 4-thiocarbonyl functionality demonstrated a dose-dependant and generally higher tube formation inhibition activity, their 4-carbonyl counterparts showed a concentration independent pattern of antiangiogenic activity. Meanwhile, the four most active compounds (**4b**, **5a**,**b** and **6a**) showed comparable levels of tube formation inhibition (~40–55% at 1 µM, 12 h) to that of the antiangiogenic drug sunitinib (50% at 1 µM, 48 h). Besides the VEGFR2 docking study, comparison of the results of the different assays used here, i.e., tube formation, migration and VEGFR2 phosphorylation activation, provided more insight into the antiangiogenic SAR of the candidate flavones. Evidently, compounds **5a**,**b** possessing 7-OH and 4-C=S groups mainly depend on VEGFR2 inhibition to mediate their observed antiangiogenic effects. In that regard, an additional OH group at position 5 resulted in higher binding affinity with VEGFR2, which translated into **5b** being more active. On the other hand, **5b**’s 5,7-dimethoxy analogue (**4b**) reliance on VEGFR2 interaction was at a much lower level. As for the flavone **6a**, no association was found between its antiangiogenic reactivity and VEGFR2. Further investigation of the docking poses of **6a** and other flavones with 4-C=O (**3a**,**b** and **6b**) with VEGFR2 showcased the negative impact of the carbonyl group on molecular interactions with the receptor. All in all, this study presented four potential antiangiogenic lead compounds (**4b**, **5a**,**b** and **6a**) and provided important perspectives on their possible mechanisms of action and SAR. These lead compounds are excellent candidates for future structural modifications and further antiangiogenic and anticancer evaluation especially since thioflavones (**5a**,**b**) have previously exhibited promising anticancer activity in vitro [36]. 

## Figures and Tables

**Table 1:** Comparison of tube formation inhibition activities of 4-C=O and 4-C=S derivatives at 10 µM.

Compound	Overall Tube Formation Inhibition at 10 µM (% of Control)
**3b** (C=O)	35% ^†^
**4b** (C=S)	66% ^†^
**6a** (C=O)	57% ^§^
**5a** (C=S)	64% ^§^
**6b** (C=O)	30% ^†^
**5b** (C=S)	60% ^†^

Statistical significance was estimated for each C=O derivative relative to its C=S counterpart by unpaired one-way *t*-test (§ *p* < 0.05, † *p* < 0.0001).

## Data Availability

Not applicable.

## Supplementary Materials

The following supporting information can be downloaded at: https://www.mdpi.com/article/10.3390/molecules27154757/s1, Figure S1: Spectral data of flavone **3a**; Figure S2: Spectral data of flavone **3b**; Figure S3: Spectral data of flavone **4a**; Figure S4: Spectral data of flavone **4b**; Figure S5: Spectral data of flavone **5a**; Figure S6: Spectral data of flavone **5b**; Figure S7: Spectral data of flavone **6a**; Figure S8: Spectral data of flavone **6b**; Table S1: Original raw western blots supporting all blot results reported in the main article; Figure S9: 2D interactions of flavonoid derivatives (**4b**, **5a**, **5b** and **6a**); Figure S10: Ribbon representation showing all 20 conformations of flavonoid derivatives (**3a**,**b**, **4b**, **5a**,**b** and **6a**,**b**) (green) compared to co-crystallized ligand (pink) in chain A of VEGFR2. Reference [36] is cited in supplementary materials.

## Sample Availability

Samples of the compounds are not available from the authors.
