# Supplementary material for: Halogenated Flavonoid Derivatives Display Antiangiogenic Activity
Source: Molecules. 2022 Jul 25;27(15):4757. doi: 10.3390/molecules27154757 (PMC9331694; doi:10.3390/molecules27154757)
Supplement: Supplementary file 1 [file molecules-27-04757-s001.zip › molecules-1799423-supplementary.pdf]

Halogenated Flavonoid Derivatives Display Antiangiogenic Activity

Supplementary Materials

1. **Chemicals and Reagents:** Chemicals, reagents and analytical grade solvents were purchased from Sigma-Aldrich unless specified. Gallacetophenone 3',4'-dimethyl ether was purchased from Apollo Scientific, UK. All reactions were carried out under an inert atmosphere and their progress was monitored using thin layer chromatograghy (TLC) which was visualized with UV lamp 254 nm. Products were purified using column chromatograghy and their purity assessed using an Agilent-1100 series HPLC equipped with Column: C-18 (ACE-221-2546; 4.6 mm x 250 mm; 5 µm) at 258 nm; 1.0 mL/min flow rate; 20 min gradient of 1% acetic acid in acetonitrile [organic mobile phase (A)] and 1% acetic acid in water [aqueous mobile phase (B)] from 5% A at 0 min, 20% A at 2 min, 40% A at 6 min, 60% A at 8 min, 95% A at 10 min, 5% A at 20 min. ^{1}H NMR and ^{13}C NMR spectra were recorded in either deuterated chloroform ($CDCl_3$) or deuterated dimethyl sulfoxide (DMSO- d_6) using a Bruker DPX 400 (400 MHz) spectrometer where chemical shifts (δ) are reported as parts per million (ppm) values relative to tetramethylsilane (TMS) as internal standard. Coupling constants (*J*) are reported in Hertz (Hz) and multiplicities are reported as follows: s (singlet) or d (doublet). Infrared spectra were recorded on a Perkin Elmer precisely spectrum 100 FT-IR spectrometer. The symbol ν indicate stretching vibrations, band intensities are classified as w, weak; m, medium; s, strong; br, broad. Mass spectrometry data were recorded on a Thermo Fisher LTQ Orbitrap XL instrument.

2. **Synthesis:**

Synthesis followed the published methods [1] to produce compounds (**1a,b** – **6a,b**).

6-Acetyl-2,3-dimethoxyphenyl 4-bromobenzoate (1a)

The pure compound was obtained as a white solid after purification by column chromatography [Hexane:$CHCl_3$:EtOAc (5:4:1 v/v/v)].
Yield: 97%; **m.p:** 101-102 °C (lit.[36] 117-119 °C); **^{1}H NMR:** ($CDCl_3$, 400 MHz) δ 2.48 (3H, s, -C**H₃**), 3.81 (3H, s, -OC**H₃**), 3.95 (3H, s, -OC**H₃**), 6.88 (1H, d, *J* = 8 Hz, H-4), 7.67 (1H, d, *J* = 8 Hz, H-5), 7.98 (2H, d, *J* = 8 Hz, H-3’,5’), 8.08 (2H, d, *J* = 8 Hz, H-2’,6’); **^{13}C NMR:** ($CDCl_3$, 100 MHz) δ 29.53 (**C**H_3), 56.20 (-O**C**H_3), 61.01 (- O**C**H_3), 109.18 (C4), 124.38 (C6), 126.16 (C5), 128.18 (C4’), 128.99 (C1’), 131.87 (C3’, C5’), 132.06 (C2’, C6’), 141.58 (C2), 144.14 (C1), 157.27 (C3), 164.10 (**C**OO-Ar), 195.66 (**C**OCH_3); **IR ν_{max} [cm^{-1}]:** 1726 (C=O, ν, s), 1670 (C=O, ν, s), 1263 (-OCH_3, ν, s), 1094 (O-C=O, ν, m); ***m/z* =** Calculated 379.0176, found 379.0171 ($C_{17}H_{16}O_5{}^{79}Br$) as $[M+H]^+$

2-Acetyl-3,5-dimethoxyphenyl 4-chlorobenzoate (1b)

The pure compound was obtained as a white solid after purification by column chromatography [Hexane:$CHCl_3$:EtOAc (6:3:1 v/v/v)].

Yield: 98%; **m.p:** 112-114 °C (lit.[36] 124-126 °C); **^{1}H NMR:** ($CDCl_3$, 400 MHz) δ 2.47 (3H, s, C**H₃**), 3.83 (3H, s, -OC**H₃**), 3.87 (3H, s, -OC**H₃**), 6.35 (1H, s, H-4), 6.41 (1H, s, H-6), 7.45 (2H, d, *J* = 8 Hz, H-3',5'), 8.06 (2H, d, *J* = 8 Hz, H- 2',6'); **^{13}C NMR:** ($CDCl_3$, 100 MHz) δ 31.98 (- **C**H_3), 55.69 (-O**C**H_3), 55.95 (-O**C**H_3), 96.76 (C6), 100.11 (C4), 117.06 (C2), 127.71 (C1'), 128.95 (C3', C5'), 131.67 (C2', C6'), 140.19 (C4'), 149.79 (C1), 159.38 (C5), 162.34 (C3), 164.26 (**C**OO-Ar), 199.16 (**C**OCH_3); **IR ν_{max} [cm^{-1}]:** 1684 (C=O, ν, s), 1609 (C=O, ν, m), 1251 (-OCH_3, ν, s), 1113 (O-C=O, s, m); ***m/z* =** Calculated 335.0681, found 335.0672 ($C_{17}H_{16}O_5{}^{35}Cl$) as $[M+H]^+$

1-(4-Bromophenyl)-3-(2-hydroxy-3,4-dimethoxyphenyl) propane-1,3- dione (2a)

Yield: 87%; **^{1}H NMR:** ($CDCl_3$, 400 MHz) [The compound exists as a mixture of keto-enol tautomers] δ 3.92 (3H, s, -OC**H₃**), 3.94 (2H, s, C**H₂**), 3.95 (3H, s, -OC**H₃**), 6.52 (1H, d, *J* = 9 Hz, H-5), 6.71 (1H, =C**H** of enol form), 7.53 (1H, d, *J* = 9 Hz, H-6), 7.61 (2H, d, *J* = 8 Hz, H-3',5'), 7.78 (2H, d, *J* = 8 Hz, H- 2',6'), 12.26 (1H, s, OH), 12.56 (1H, s, OH of enol form).

1-(4-Chlorophenyl)-3-(2-hydroxy-4,6-dimethoxyphenyl) propane-1,3- dione (2b)

Yield: 80%; **^{1}H NMR:** ($CDCl_3$, 400 MHz) [The compound exists as a mixture of keto-enol tautomers] δ 3.47 (3H, s, -OC**H₃**), 3.88 (3H, s, -OC**H₃**), 4.68 (2H, s, C**H₂**), 6.10 (1H, s, H-5), 6.20 (1H, s, H-3), 7.20 (1H, s, -C**H** of enol form), 7.63 (2H, d, *J* = 8 Hz, H-3',5'), 7.99 (2H, d, *J* = 8 Hz, H- 2',6'), 13.24 (1H, s, OH), 13.65 (1H, s, OH of enol form).

2-(4-Bromophenyl)-7,8-dimethoxy-4H-chromen-4-one (3a)

The pure compound was obtained as a white solid after purification by column chromatography [$CHCl_3$: Hexane: EtOAc (5:3:2 v/v/v)].

Yield: 88%; **m.p:** 198-198.5 °C (lit.[36] 197-199 °C); **^{1}H NMR:** ($CDCl_3$, 400 MHz) δ 4.01 (3H, s, -OC**H₃**), 4.03 (3H, s, -OC**H₃**), 6.75 (1H, s, H-3), 7.05 (1H, d, *J* = 9 Hz, H-6), 7.66 (2H, d, *J* = 8 Hz, H-2',6'), 7.81 (2H, d, *J* = 8 Hz, H-3',5'), 7.95 (1H, d, *J* = 9 Hz, H-5); **^{13}C NMR:** ($CDCl_3$, 100 MHz) δ 56.47 (- O**C**H_3), 61.63 (-O**C**H_3), 107.06 (C3), 110.06 (C6), 118.62 (C10), 121.12 (C5), 126.24 (C4'), 127.65 (C2', C6'), 130.93 (C1), 132.40 (C3', C5'), 136.97 (C8), 150.52 (C9), 156.82 (C7), 161.95 (C2), 177.95 (**C=**O, C4); **IR ν_{max} [cm^{-1}]:** 1641 (C=O, ν, m), 1288 (-OCH_3, ν, s) 1093 (C-O, ν, m); ***m/z* =** Calculated 361.0070, found 361.0066 ($C_{17}H_{14}O_4{}^{79}Br$) as $[M+H]^+$. HPLC Purity: > 99.4% (R_t= 13.59 min)

2-(4-Chlorophenyl)-5,7-dimethoxy-4H-chromen-4-one (3b)

The pure compound was obtained as a white solid after purification by column chromatography [EtOAc (100%) followed by 5% MeOH in EtOAc].

Yield: 77%; **m.p:** 174-175 °C (lit.[36] 188-192 °C); **^{1}H NMR:** ($CDCl_3$, 400 MHz) δ 3.91 (3H, s, -OC**H₃**), 3.95 (3H, s,-OC**H₃**), 6.37 (1H, s, H-6), 6.55 (1H, s, H-8), 6.64 (1H, s, H-3), 7.45 (2H, d, *J* = 8 Hz, H-2',6'), 7.78 (2H, d, *J* = 8 Hz, H-3',5'); **^{13}C NMR:** ($CDCl_3$, 100 MHz) δ 55.79 (-O**C**H_3), 56.43 (-O**C**H_3),

92.79 (C8), 96.26 (C6), 109.14 (C3), 127.17 (C3′,5′,1′), 129.23 (C2′, C6′), 129.97 (C10), 137.36 (C4′), 159.47 (C9), 159.78 (C5), 160.91 (C2), 164.16 (C7), 177.38 (**C=**O); **IR *ν*max [cm⁻¹]:** 1673 (C=O, v, s), 1175 (-OCH_3, v, m), 1110 (C-O, v, m); ***m/z* =** Calculated 317.0575, found 317.0573 ($C_{17}H_{14}O_4{}^{35}Cl$) as $[M+H]^+$. HPLC Purity: > 96.4% (R_t= 13.19 min)

2-(4-Bromophenyl)-7,8-dimethoxy-4H-chromene-4-thione (4a)

The pure compound was obtained as an orange solid after purification by column chromatography [$CHCl_3$ (100%)].

Yield: 88% ; **m.p:** 214-216 °C (lit.[36] 216-218 °C); **^{1}H NMR:** ($CDCl_3$, 400 MHz) δ 4.02 (3H, s, -OC**H₃**), 4.03 (3H, s, -OC**H₃**), 7.07 (1H, d, *J* = 9.5 Hz, H-6), 7.66 (1H, s, H-3), 7.67 (2H, d, *J* = 8.5 Hz, H-2′,6′), 7.87 (2H, d, *J* = 8 Hz, H- 3′,5′), 8.33 (1H, d, *J* = 9 Hz, H-5); **^{13}C NMR:** ($CDCl_3$, 100 MHz) δ 56.54 (-O**C**H_3), 61.74 (- O**C**H_3), 111.14 (C6), 119.08 (C3), 124.19 (C5, C10), 125.52 (C4′), 126.56 (C1′), 127.88 (C2′, C6′), 132.59 (C3′, C5′), 137.27 (C8), 145.90 (C9), 152.41 (C7), 157.39 (C2), 201.33 (**C**=S); **IR *ν*max [cm⁻¹]:** 1288 (C=S, v, s), 1091 (-OCH_3, v, m), 1040 (C-O, v, s); ***m/z* =** Calculated 376.9842, found 376.9840 ($C_{17}H_{14}O_3{}^{79}BrS$) as $[M+H]^+$. HPLC Purity: > 92.3% (R_t= 13.36 min)

2-(4-Chlorophenyl)-5,7-dimethoxy-4H-chromene-4-thione (4b)

The pure compound was obtained as a green solid after purification by column chromatography [$CHCl_3$ (100%)].

Yield: 77%; **m.p:** 173.1-173.8 °C (lit.[36] 170-172 °C); **^{1}H NMR:** ($CDCl_3$, 400 MHz) δ 3.91 (3H, s, -OC**H₃**), 3.93 (3H, s, -OC**H₃**), 6.40 (1H, d, *J* = 2 Hz, H-6), 6.55 (1H, d, *J* = 2 Hz, H-8), 7.44 (2H, d, *J* = 8 Hz, H-2′,6′), 7.48 (1H, s, H-3), 7.81 (2H, d, *J* = 8.5 Hz, H- 3′,5′); **^{13}C NMR:** ($CDCl_3$, 100 MHz) δ 55.87 (-O**C**H_3), 55.89 (- O**C**H_3), 92.73 (C8), 96.90 (C6), 117.83 (C10), 122.30 (C3), 127.30 (C2′, 6′), 129.30 (C3′, 5′), 129.48 (C1′), 137.44 (C4′), 148.49 (C2), 155.59 (C9), 161.56 (C7), 163.94 (C5), 200.36 (**C**=S); **IR *ν*max [cm⁻¹]:** 1295 (C=S, v, s), 1091 (-OCH_3, v, m), 1044 (C-O, v, s); ***m/z* =** Calculated 333.0347 found 333.0344 ($C_{17}H_{14}O_3{}^{35}ClS$) as $[M+H]^+$. HPLC Purity: > 93.5 % (R_t= 14.22 min)

2-(4-Bromophenyl)-7,8-dihydroxy-4H-chromene-4-thione (5a)

The pure compound was obtained as an orange solid after purification by column chromatography [EtOAc (100%)].
Yield: 85%; **m.p:** 223.8-224 °C (lit.[36] 235-237 °C); **^{1}H NMR:** (DMSO- d_6, 400 MHz) δ 6.98 (1H, d, *J* = 8.5 Hz, H-6), 7.70 (1H, s, H-3), 7.74 (2H, d, *J* = 8 Hz, H-2′,6′), 7.77 (2H, d, *J* = 9 Hz, H-3′,5′), 8.12 (1H, d, *J* = 8.5 Hz, H-5), 9.62 (1H, s, OH), 10.57 (1H, s, OH); **^{13}C NMR:** (DMSO- d_6, 100 MHz) δ 116.23 (C6), 118.25 (C4′), 119.00 (C3), 124.44 (C5), 126.14 (C10), 129.22 (C2′, C6′), 130.54 (C1′), 132.69 (C3′, C5′), 133.53 (C8), 142.85 (C7), 152.27 (C9), 152.69 (C2), 200.36 (**C=**S); **IR *ν*max [cm⁻¹]:** 3501 (OH, v, s), 1279 (C=S, v, s), 1174 (C-O, v, m); ***m/z* =** Calculated 348.9529, found 348.9529 ($C_{15}H_{10}O_3{}^{79}BrS$) as $[M+H]^+$. HPLC Purity: > 90.5%

2-(4-Chlorophenyl)-5,7-dihydroxy-4H-chromene-4-thione (5b)

The pure compound was obtained as a bright yellow solid after purification by column chromatography [$CHCl_3$:Hexane:EtOAc (8:1:1) v/v/v)].

Yield: 64%; **m.p:** 247.7-248 °C (lit.[36] 249-252 °C); **^{1}H NMR:** (DMSO- d_6, 400 MHz) δ 6.28 (1H, s, H-6), 6.55 (1H, s, H-8), 7.55 (1H, s, H-3), 7.58 (2H, d, *J* = 8 Hz, H-2',6'), 8.11 (2H, d, *J* = 8 Hz, H-3',5'), 11.24 (1H, s, OH), 13.54 (1H, s, OH); **^{13}C NMR:** (DMSO- d_6, 100 MHz) δ 95.28 (C8), 101.30 (C6), 113.13 (C10), 118.00 (C3), 129.84 (C1', 3', 5'), 129.84 (C2', C6'), 137.63 (C4'), 153.62 (C2), 154.55 (C9), 162.32 (C7), 165.19 (C5), 196.52 (**C=**S); **IR ν_{max} [cm^{-1}]:** 3358 (OH, w, b), 1170 (C=S, v, m), 1135 (c-O, v, m); ***m/z* =** Calculated 305.0034, found 305.0034 ($C_{15}H_{10}O_3{}^{35}ClS$) as $[M+H]^+$. HPLC Purity: > 98.6% (R_t= 14.17 min)

2-(4-Bromophenyl)-7,8-dihydroxy-4H-chromen-4-one (6a)

The pure compound was obtained as a pale green solid after purification by column chromatography [EtOAc (100%)].

Yield: 60%; **m.p:** 275-280 °C (lit.[36] 285-290 °C); **^{1}H NMR:** (DMSO- d_6, 400 MHz) δ 6.99 (1H, s, H-3), 7.00 (1H, d, *J* = 8.5 Hz, H-6), 7.44 (1H, d, *J* = 8.5 Hz, H-5), 7.84 (2H, d, *J* = 8 Hz, H-2',6'), 8.16 (2H, d, *J* = 8.5 Hz, H-3',5'); **^{13}C NMR:** (DMSO- d_6, 100 MHz) δ 106.32 (C3), 114.10 (C6), 115.08 (C5), 116.84 (C10), 125.16 (C4'), 128.29 (C2', C6'), 130.70 (C1'), 131.98 (C3', C5'), 133.07 (C8), 146.50 (C9), 150.62 (C7), 160.61 (C2), 176.77 (**C=**O); **IR ν_{max} [cm^{-1}]:** 3365 (OH, w, b), 1618 (C=O, v, m), 1066 (C-O, v, s); ***m/z* =** Calculated 332.9757, found 332.9754 ($C_{15}H_{10}O_4{}^{79}Br$) as $[M+H]^+$. HPLC Purity: > 98.8% (R_t= 11.22 min)

2-(4-Chlorophenyl)-5,7-dihydroxy-4H-chromen-4-one (6b)

The pure compound was obtained as a pale yellow solid after purification by column chromatography [EtOAc (100%)].

Yield: 97%; **m.p:** 250 °C (lit.[36] 294-296 °C); **^{1}H NMR:** (DMSO- d_6, 400 MHz) δ 6.29 (1H, s, H-6), 6.59 (1H, s, H-8), 7.08 (1H, s, H-3), 7.62 (2H, d, *J* = 8.5 Hz, H-2',6'), 7.99 (2H, d, *J* = 8.5 Hz, H-3',5'), 12.20 (1H, s, OH), 12.84 (1H, s, OH); **^{13}C NMR:** (DMSO- d_6, 100 MHz) δ 93.98 (C8), 99.04 (C6), 105.51 (C3, C10), 128.22 (C1'), 128.70 (C3', C5'), 129.18 (C2', C6'), 131.93 (C4'), 157.36 (C9), 161.39 (C5), 161.94 (C2), 166.41 (C7), 181.83 (**C=**O); **IR ν_{max} [cm^{-1}]:** 3350 (OH, w, b), 1680 (C=O, v, m), 1091 (C-O, v, s); ***m/z* =** Calculated 289.0262, found 289.0260 ($C_{15}H_{10}O_4{}^{35}Cl$) as $[M+H]^+$. HPLC Purity: > 99.4% (R_t= 13.06 min)

(A)

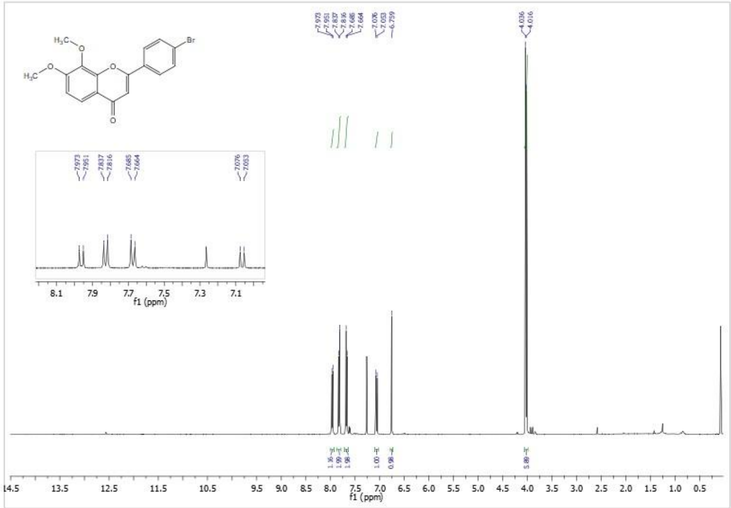

(B)

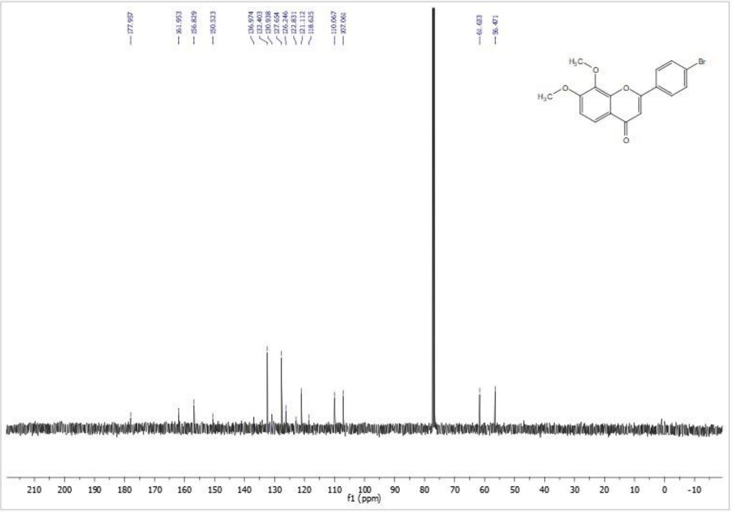

(C)

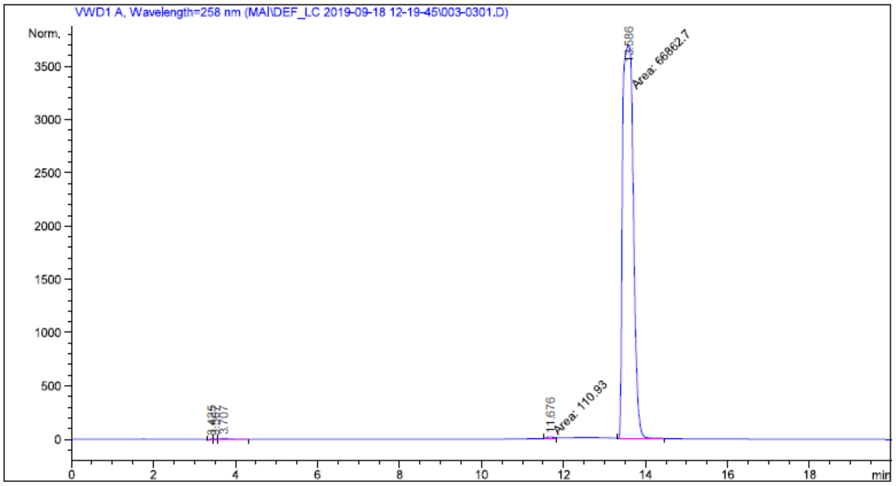

Figure S1. *Spectral data of flavone 3a. (A) ^{1}H NMR; (B) ^{13}C NMR; (C) HPLC*

(A)

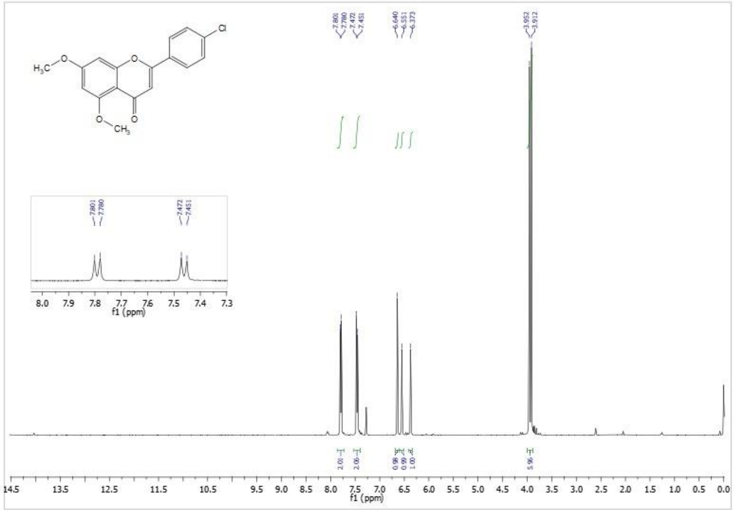

(B)

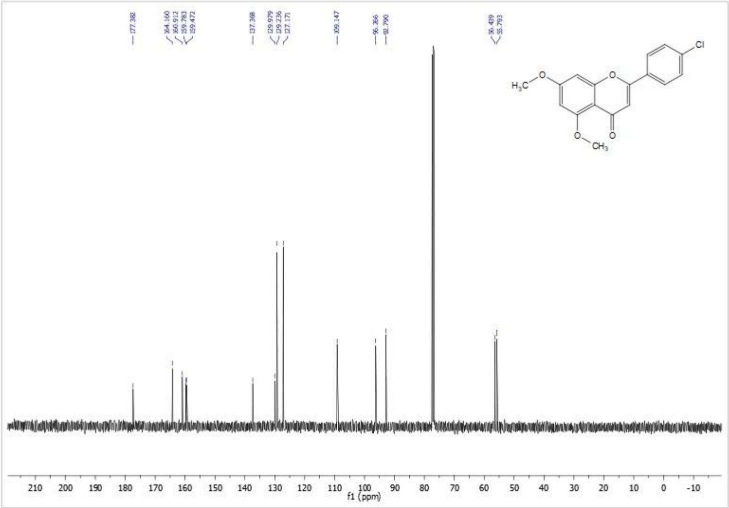

(C)

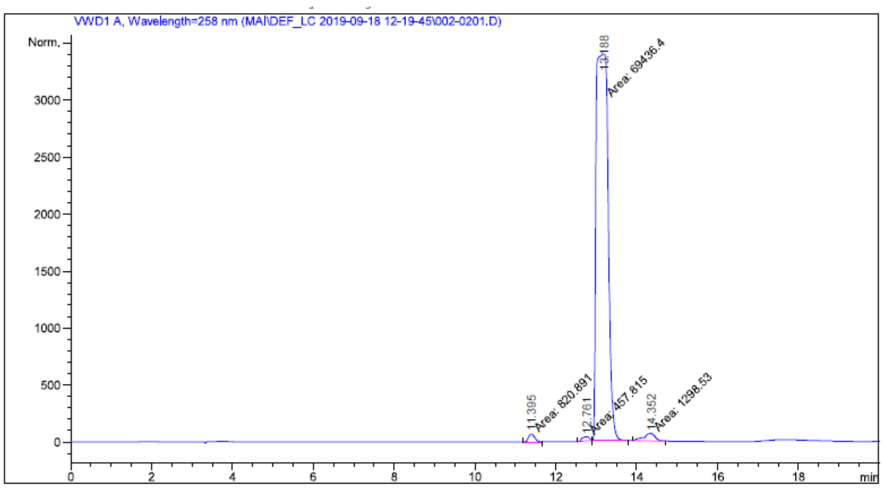

Figure S2. *Spectral data of flavone 3b. (A)* ^{1}*H NMR; (B)* ^{13}*C NMR; (C) HPLC*

(A)

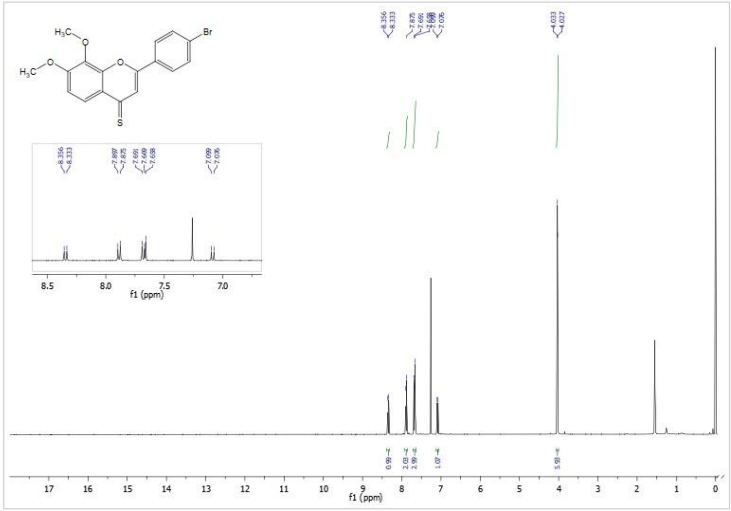

(B)

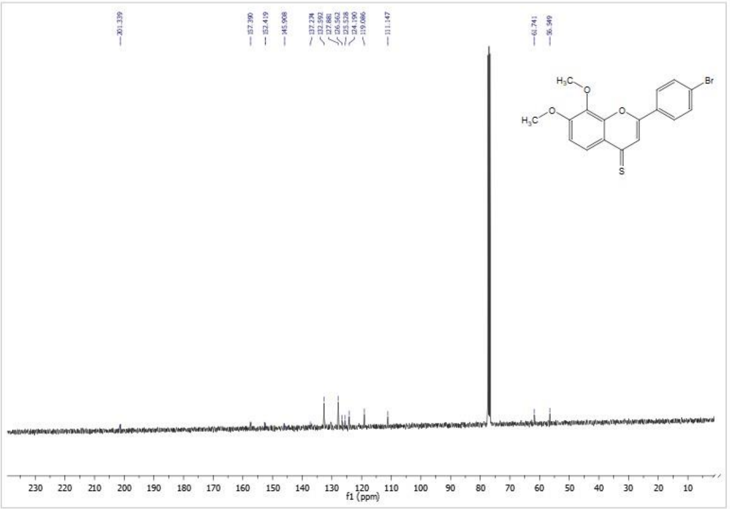

(C)

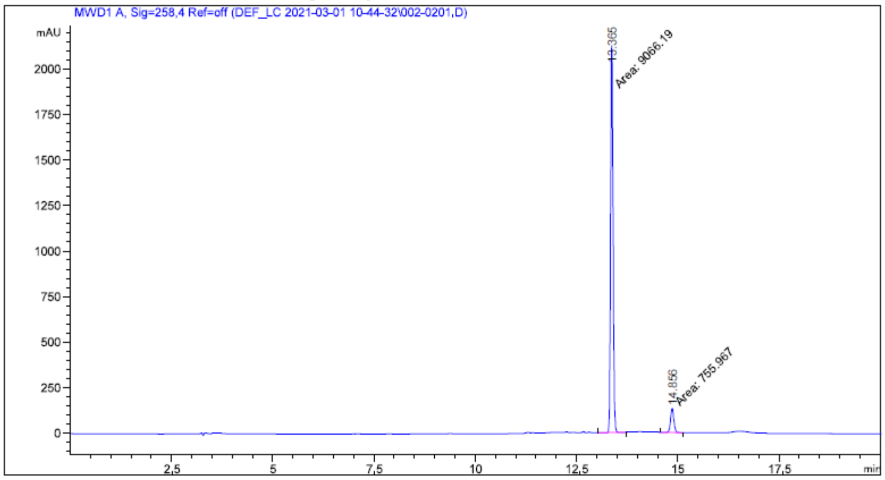

Figure S3. *Spectral data of flavone 4a. (A) ^{1}H NMR; (B) ^{13}C NMR; (C) HPLC*

(A)

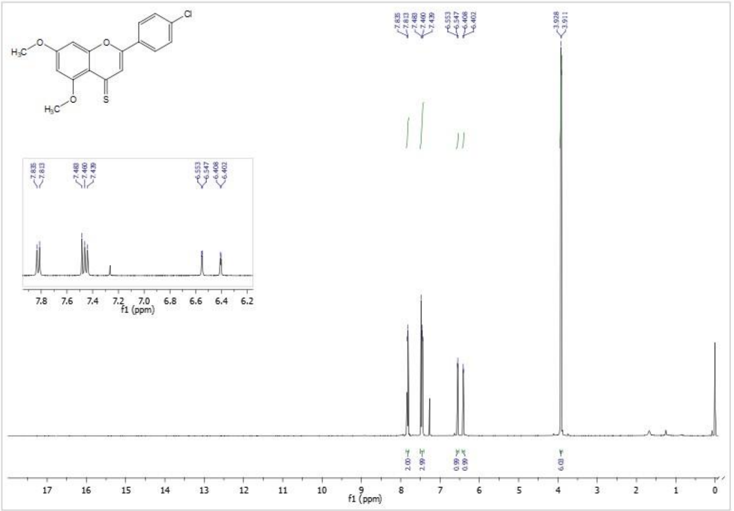

(B)

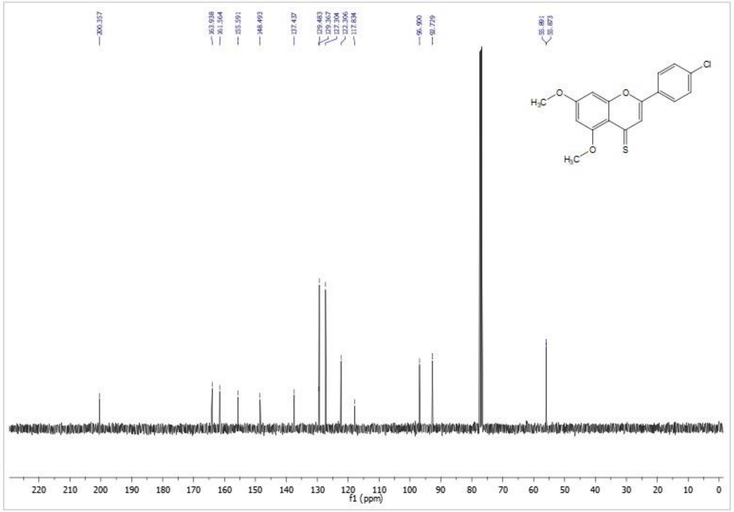

(C)

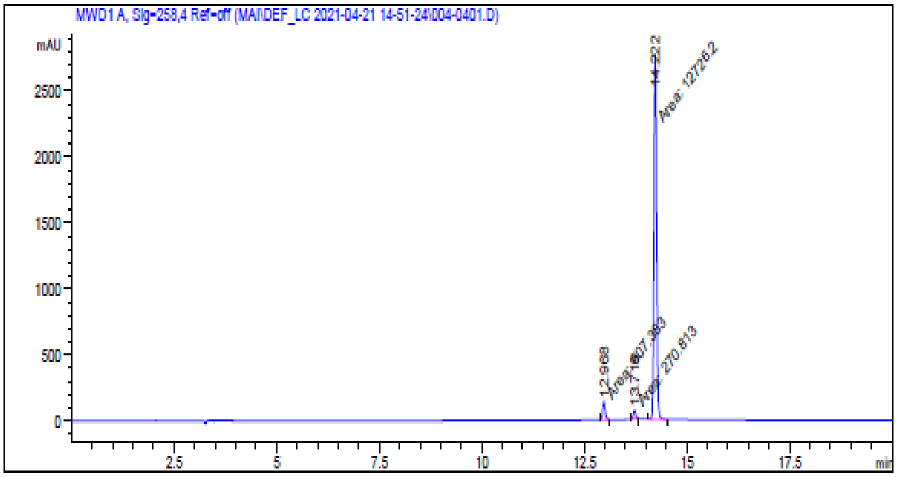

Figure S4. *Spectral data of flavone 4b. (A) ^{1}H NMR; (B) ^{13}C NMR; (C) HPLC*

(A)

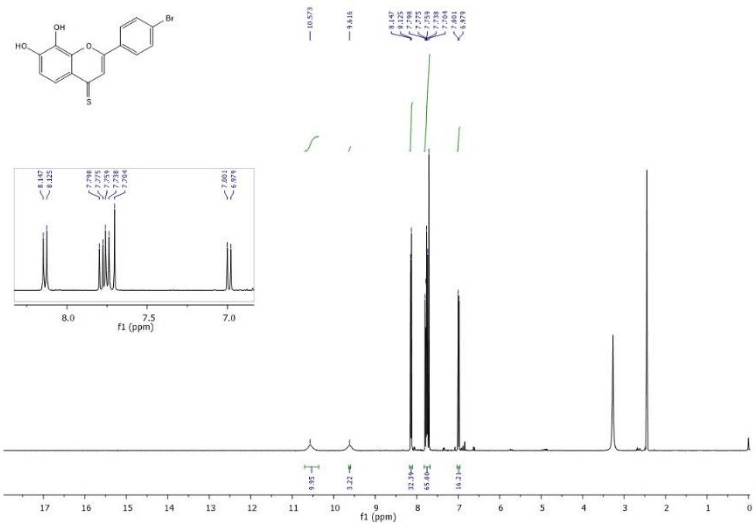

(B)

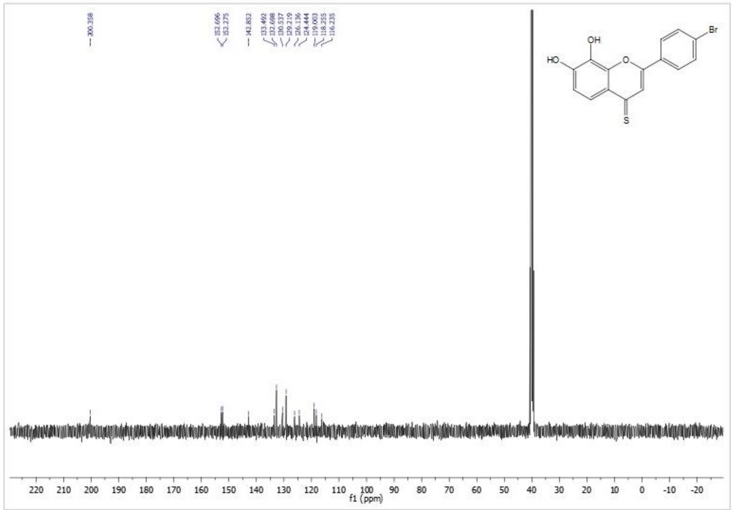

(C)

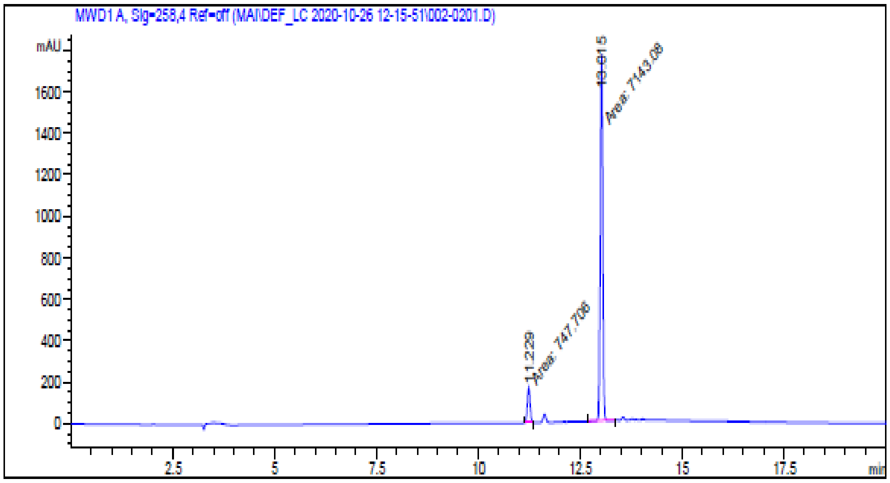

Figure S5. *Spectral data of flavone 5a. (A) ^{1}H NMR; (B) ^{13}C NMR; (C) HPLC*

(A)

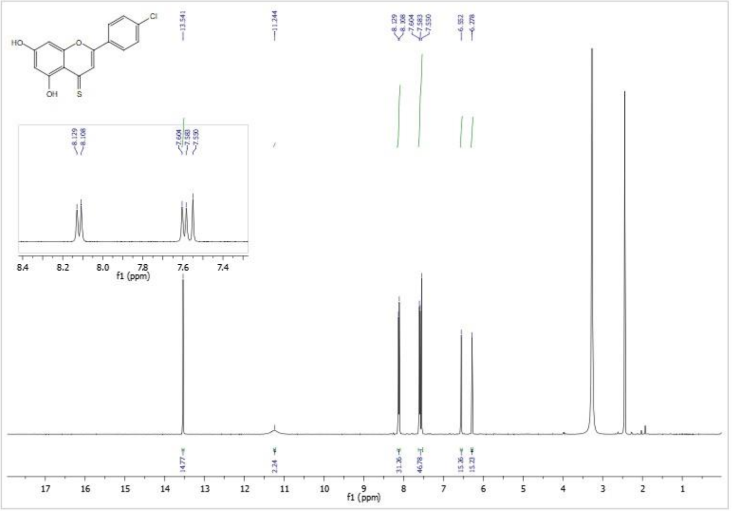

(B)

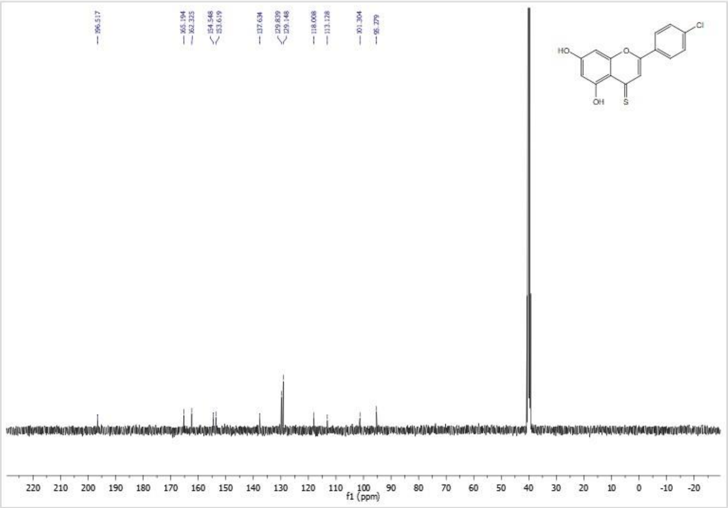

(C)

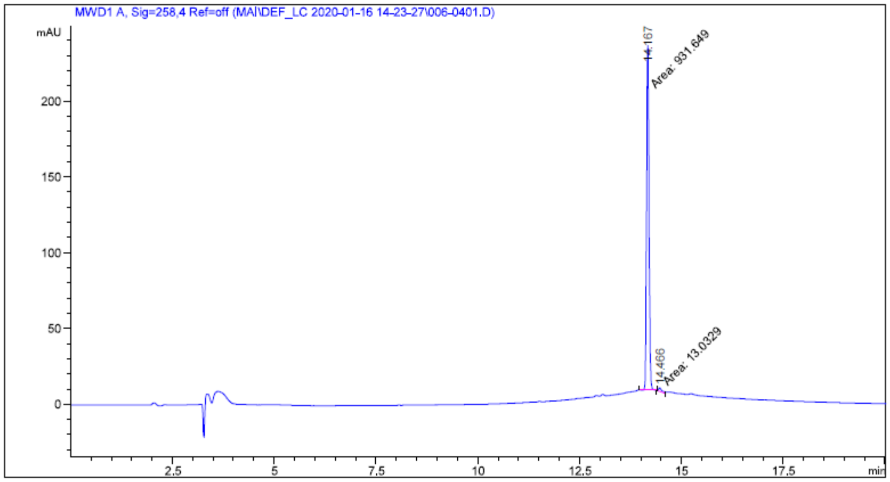

Figure S6. *Spectral data of flavone 5b. (A) ^{1}H NMR; (B) ^{13}C NMR; (C) HPLC*

(A)

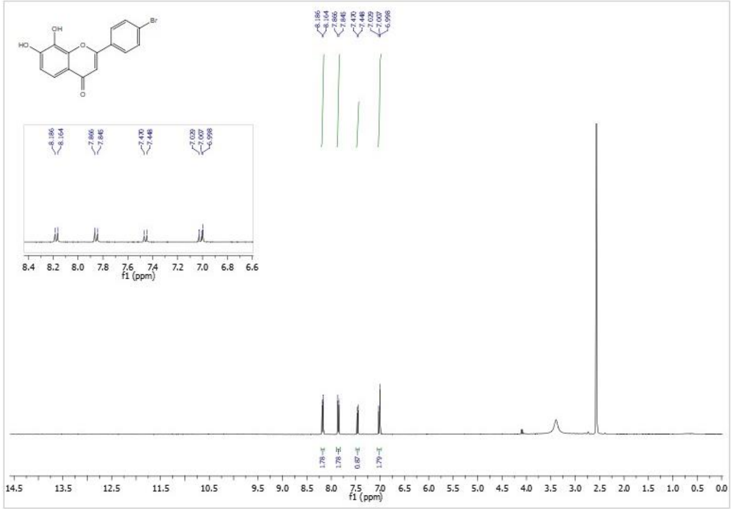

(B)

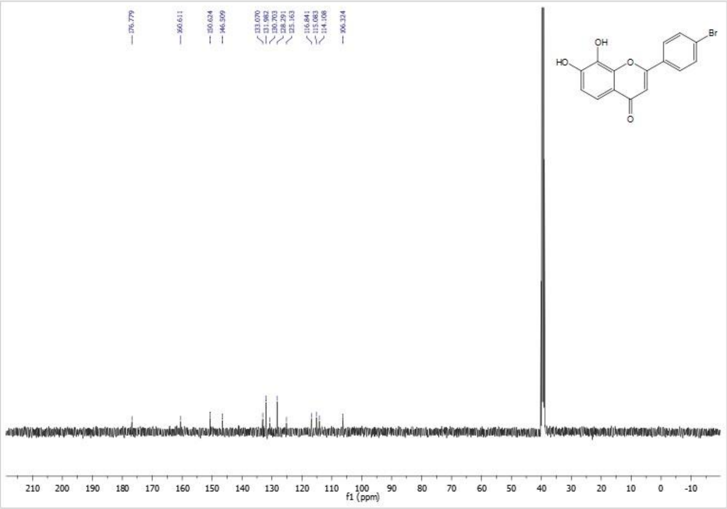

(C)

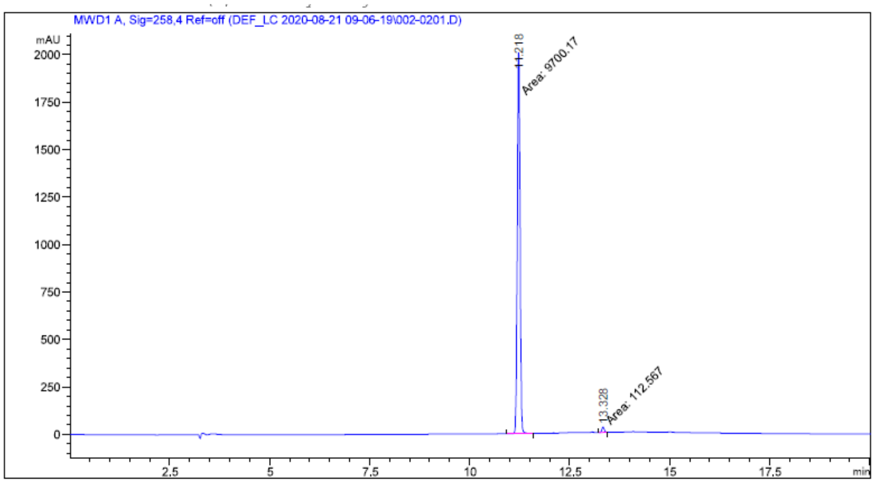

Figure S7. *Spectral data of flavone 6a. (A) ^{1}H NMR; (B) ^{13}C NMR; (C) HPLC*

(A)

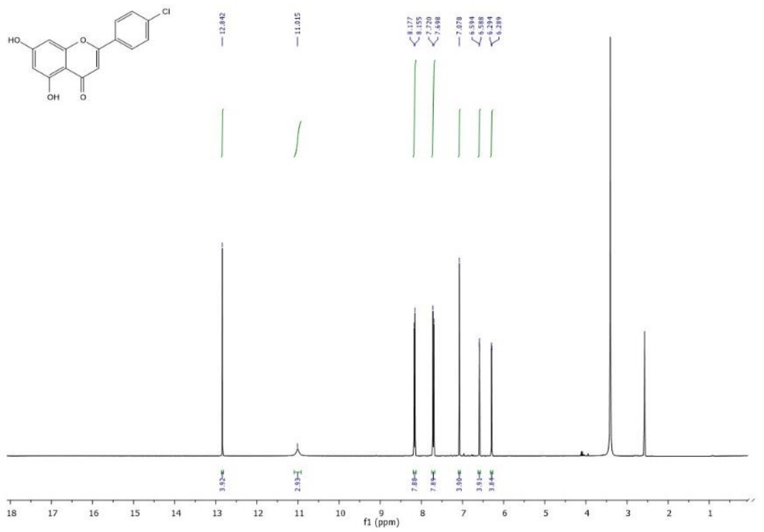

(B)

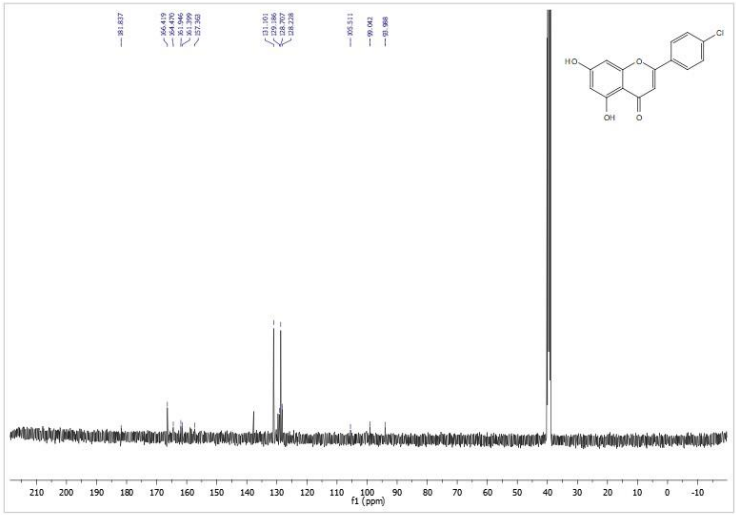

(C)

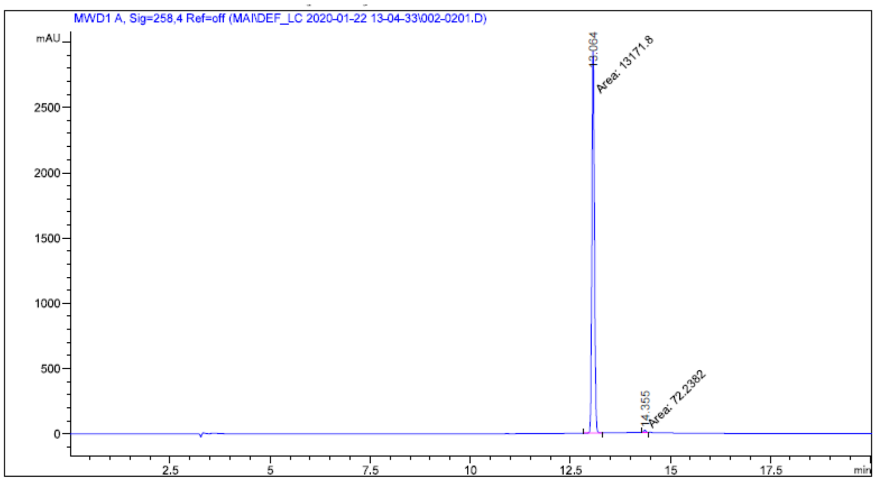

Figure S8. *Spectral data of flavone 6b. (A) ^{1}H NMR; (B) ^{13}C NMR; (C) HPLC*

3. Western blotting:

Table S1. Original raw western blots supporting all blot results reported in the main article.

	n1	n2	n3
Compound<br>Concentration (µM)	**+veC 4b 4b 5a 5a 5b 5b 6a 6a**<br>**10 1 10 1 10 1 10 1**	**+veC 4b 4b 5a 5a 5b 5b 6a 6a**<br>**10 1 10 1 10 1 10 1**	**5b 5b 4b 4b 5a 5a 6a 6a +veC**<br>**10 1 1 10 1 10 1 10**
T-VEGFR2	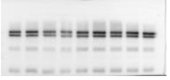	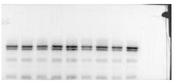	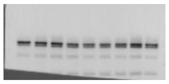
P-VEGFR2	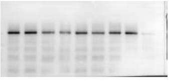	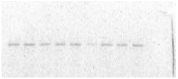	
β-Actin	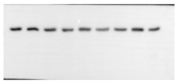	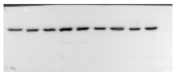	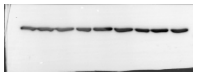

4. Molecular docking:

(A)

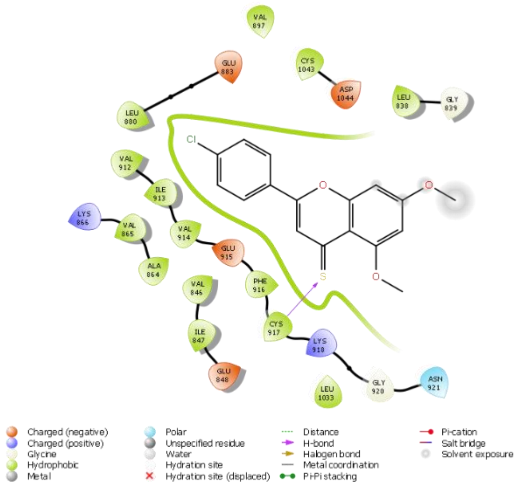

(B)

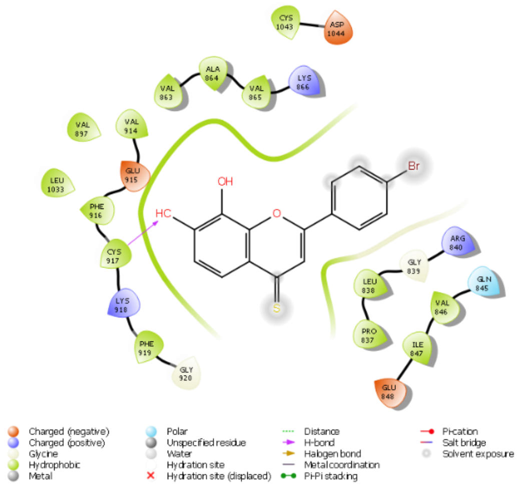

(C)

(D)

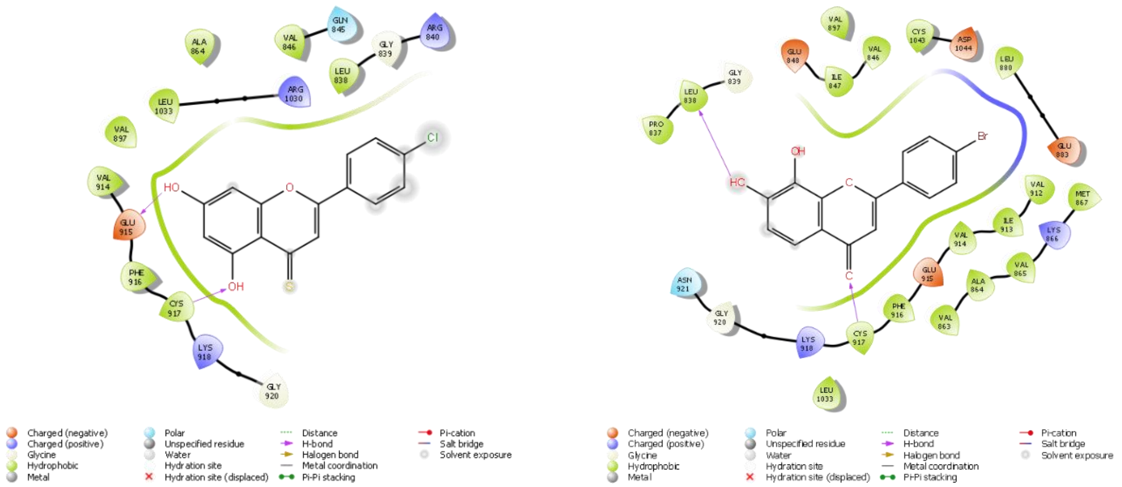

Figure 9. 2D interactions of flavonoid derivatives (4b, 5a, 5b and 6a). (A) 4b; (B) 5a; (C) 5b; (D) 6a.

(A)

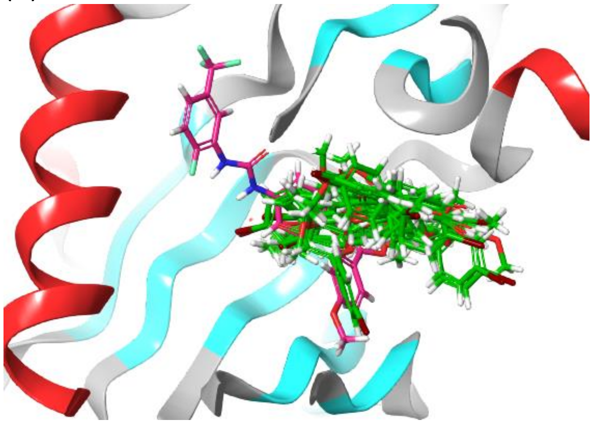

(B)

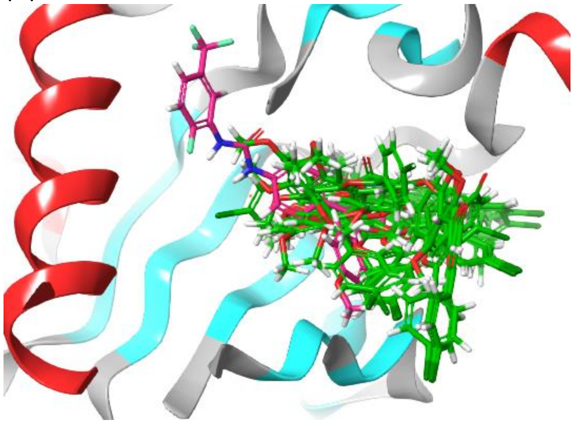

(C)

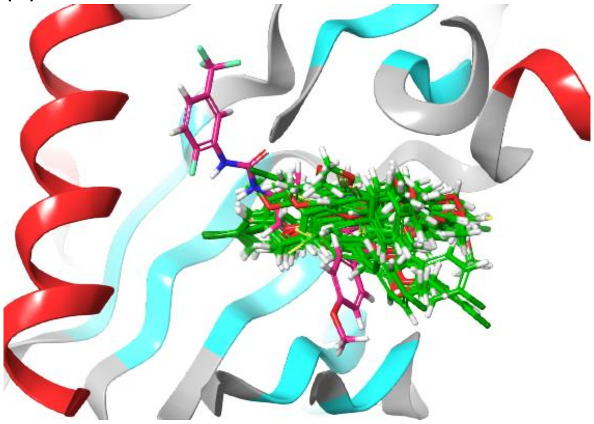

(D)

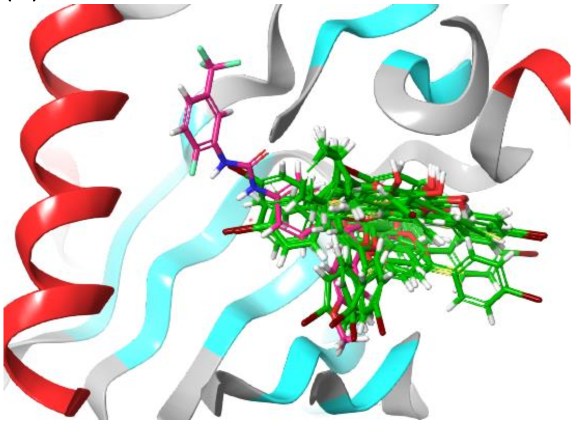

(E)

(F)

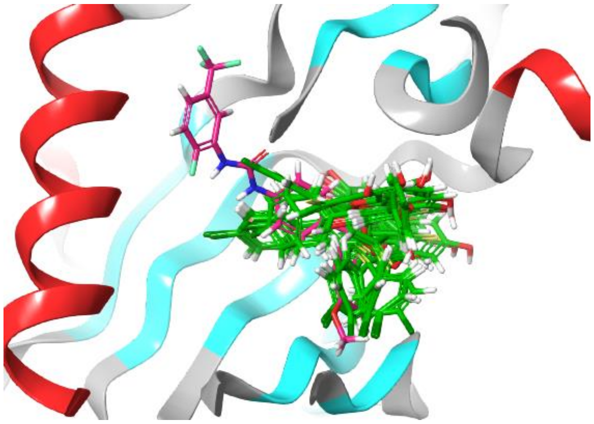

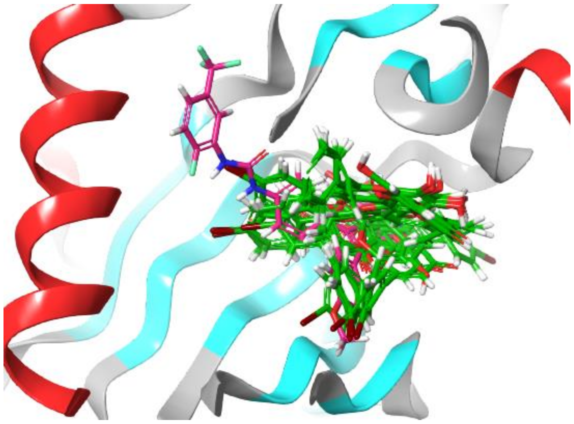

(G)

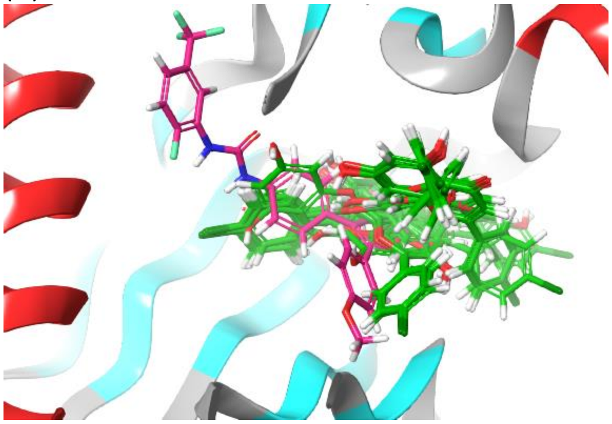

Figure S10. Ribbon representation showing all 20 conformations of flavonoid derivatives (3a,b, 4b, 5a,b and 6a,b) (green) compared to co-crystallized ligand (pink) in chain A of VEGFR2. (A) 3a; (B), 3b; (C) 4b; (D), 5a; (E) 5b; (F) 6a; (G) 6b.
